# Supplementary figures and images for: Age-related changes in tau and autophagy in human brain in the absence of neurodegeneration
Source: PLoS One. 2023 Jan 26;18(1):e0262792. doi: 10.1371/journal.pone.0262792 (PMC9879510; doi:10.1371/journal.pone.0262792)

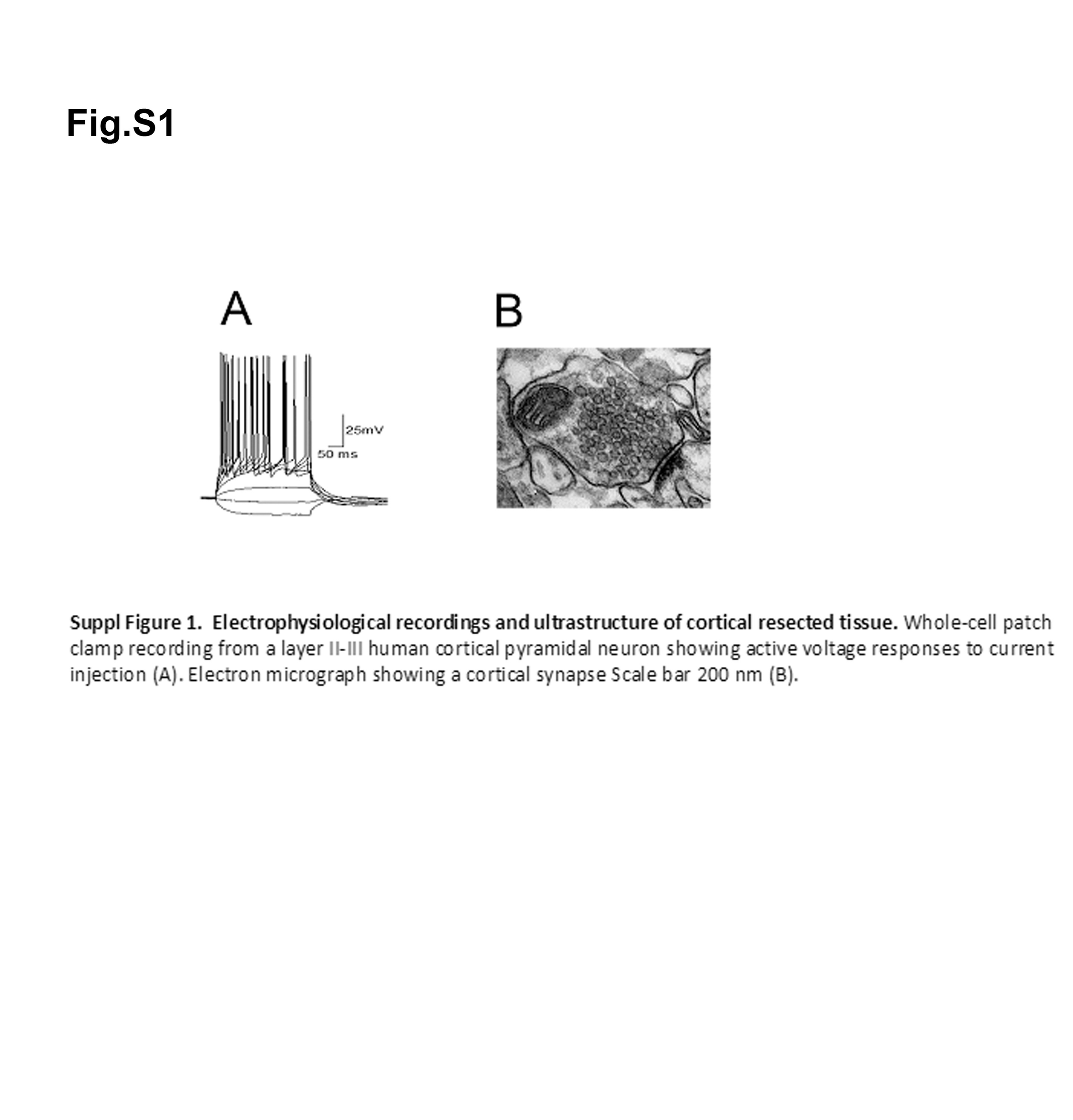

Supplement: S1 Fig — Whole-cell patch clamp recording from a layer II-III human cortical pyramidal neuron showing active voltage responses to current injection (A). Electron micrograph showing a cortical synapse. Scale Bar 200 nm (B). (TIF) [file pone.0262792.s001.tif]

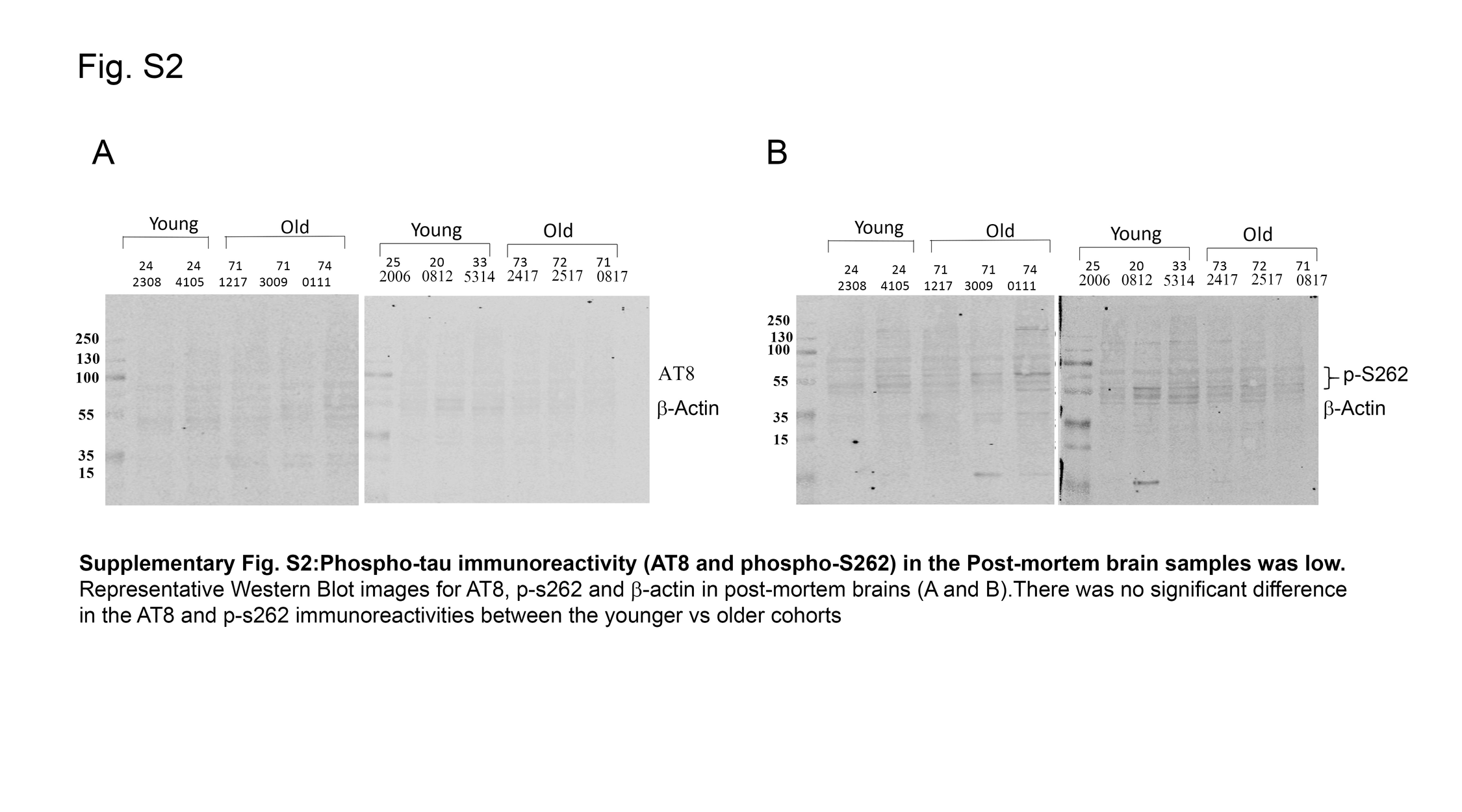

Supplement: S2 Fig — Representative Western Blot images for AT8, ps-262 and β-actin in post-mortem brains (A and B). There was no significant difference in the AT8 and ps-262 immunoreactivities between the younger vs older cohorts. (TIF) [file pone.0262792.s002.tif]

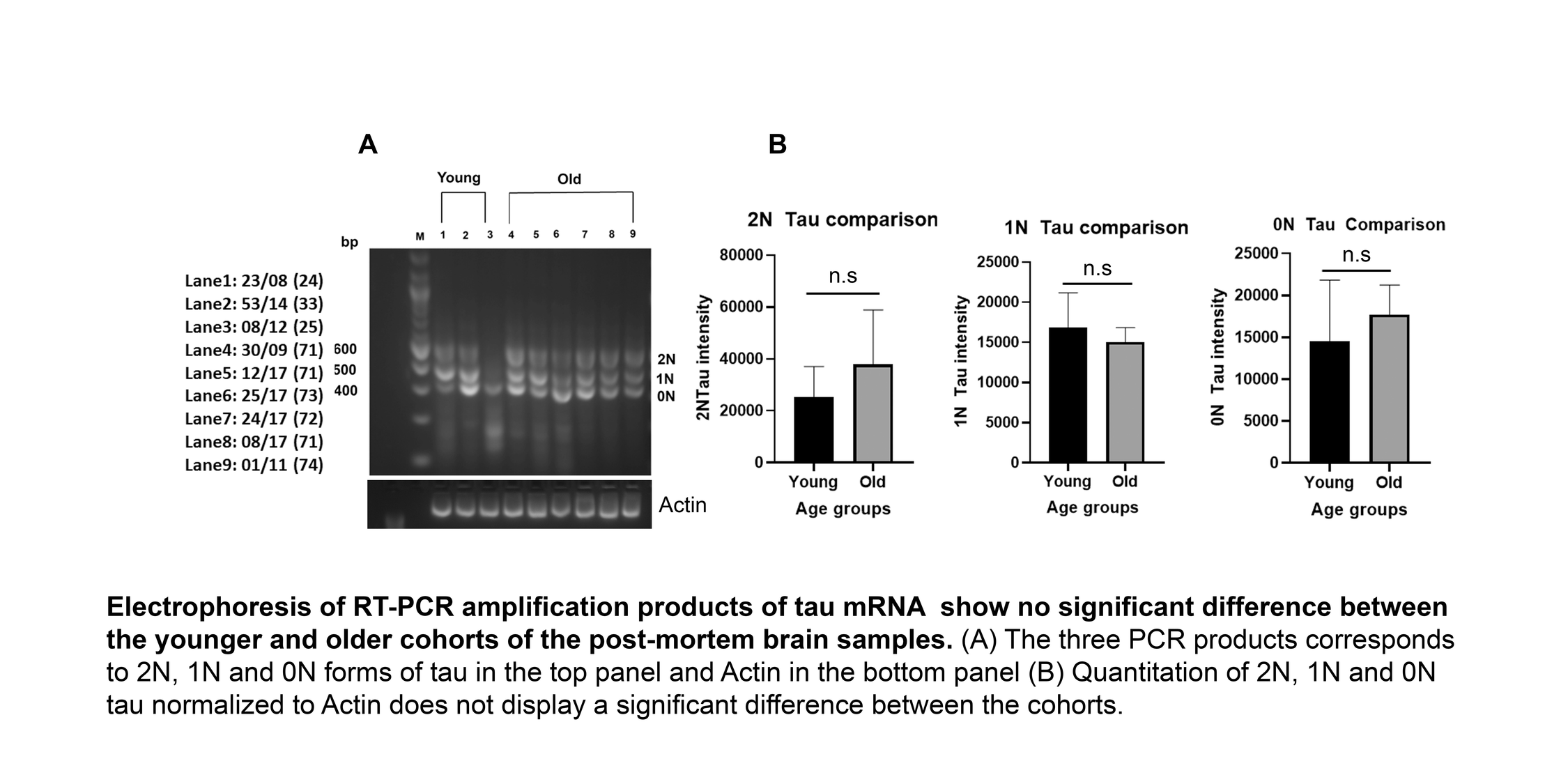

Supplement: S3 Fig — The three PCR products correspond to the 2N, 1N and 0N forms of tau in the top panel and Actin in the bottom panel (A). Quantitation of 2N, 1N and 0N tau normalized to Actin does not display a significant difference between the cohorts (B). (TIF) [file pone.0262792.s003.tif]

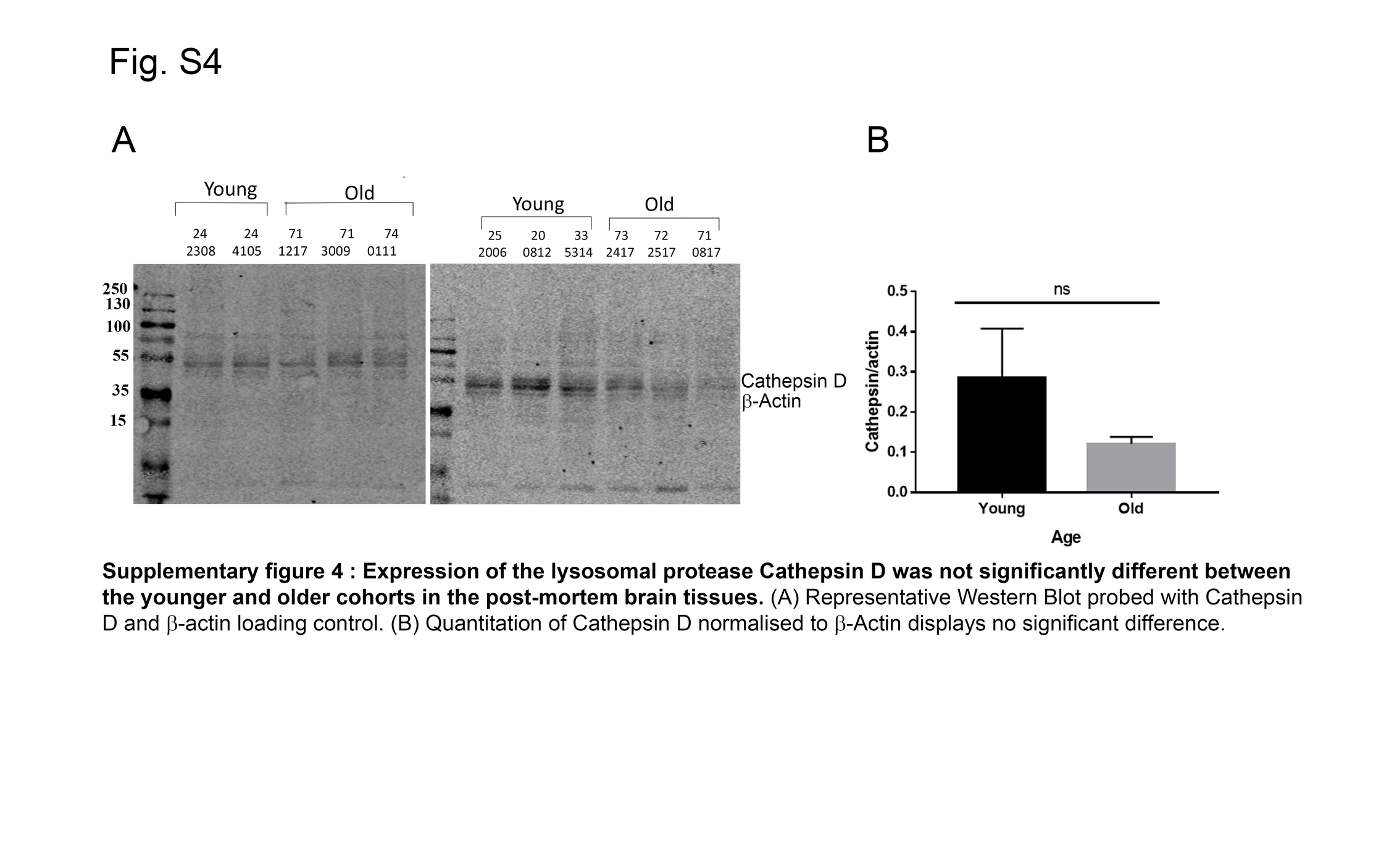

Supplement: S4 Fig — Representative western blot probed with Cathepsin D and b-actin loading control (A). Quantitation of Cathepsin D normalised to β-Actin displays no significant difference. (TIF) [file pone.0262792.s004.tif]

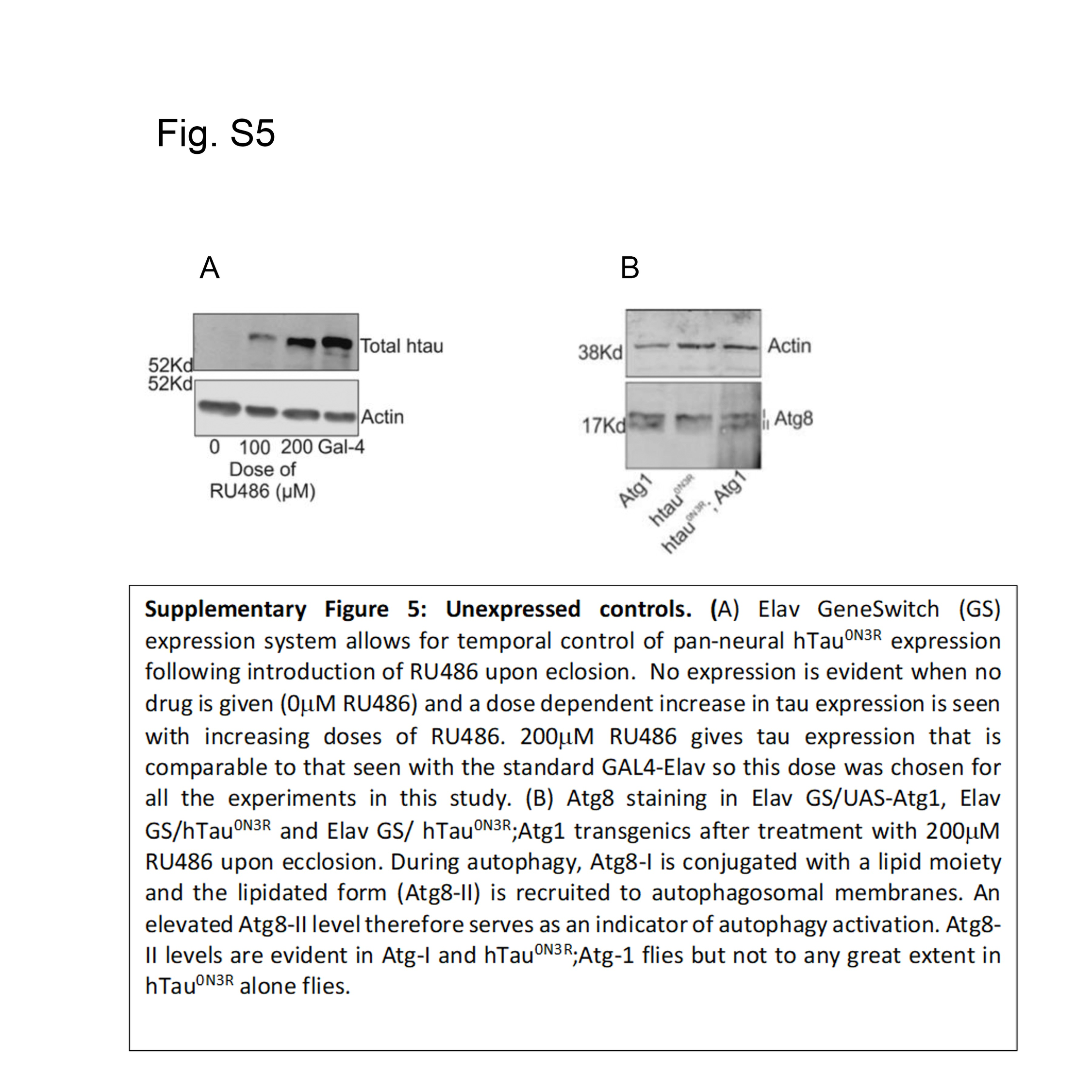

Supplement: S5 Fig — Elav Geneswitch (GS) expression system allows for temporal control of pan-neural hTau0N3R expression following introduction of RU486 upon eclosion. No expression is evident when no drug is given (0μm RU486) and a dose dependent increase in tau expression is seen with increasing doses of RU486. 200 μM RU486 gives tau expression that is comparable to that seen with the standard Elav Gal4 so this dose was chosen for all the experiments in this study (A). Atg8 staining in Elav GS/UAS-Atg1, Elav GS/hTau0N3R and Elav GS/htau0N3R;Atg1 transgenics after treatment with 200 μM RU486 upon eclosion. During autophagy, Atg8-I is conjugated with a lipid moiety and the lapidated form (Atg8-II) is recruited to the autophagosomal membranes. An elevated Atg8-II level therefore serves as an indicator of autophagy activation. Atg8-II levels are evident in the Atg1 and hTau0N3R;Atg1 flies but not to that extent in the hTau0N3R alone flies (B). (TIF) [file pone.0262792.s005.tif]

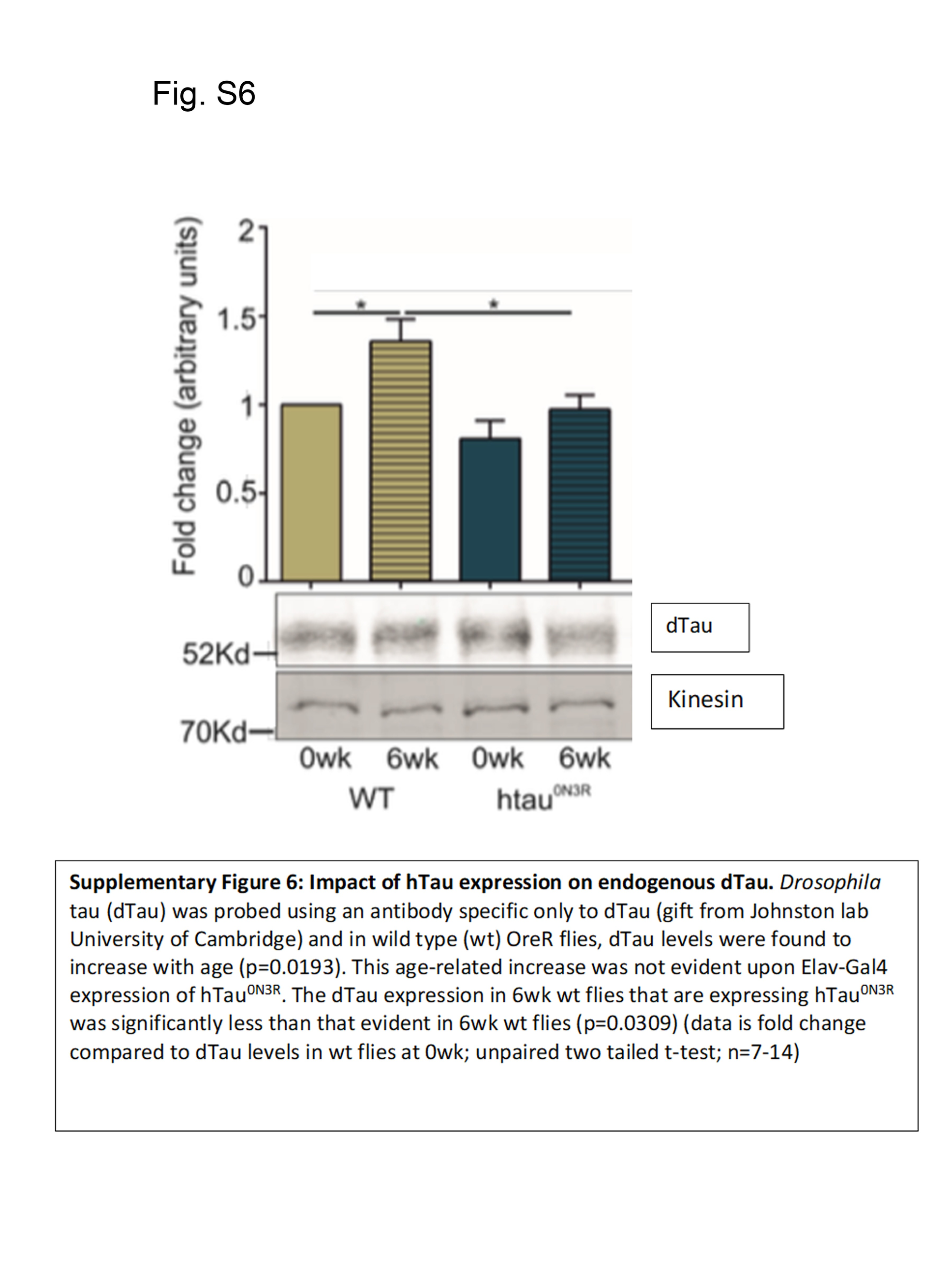

Supplement: S6 Fig — Drosophila tau (dTau) was probed using an antibody specific only to dTau (gift from Johnston lab University of Cambridge) and in wild type (wt) OreR flies, dTau levels were found to increase with age (p = 0.0193). This age-related increase was not evident upon Elav Gal4 expression of hTau0N3R. The dTau expression in 6 week wt flies that are expressing hTau0N3R was significantly less than that evident in 6 week wt flies (p = 0,0309) (data is fold change compared to dTau levels in wt flies at 0 week; unpaired two tailed t-test; n = 7–14). (TIF) [file pone.0262792.s006.tif]

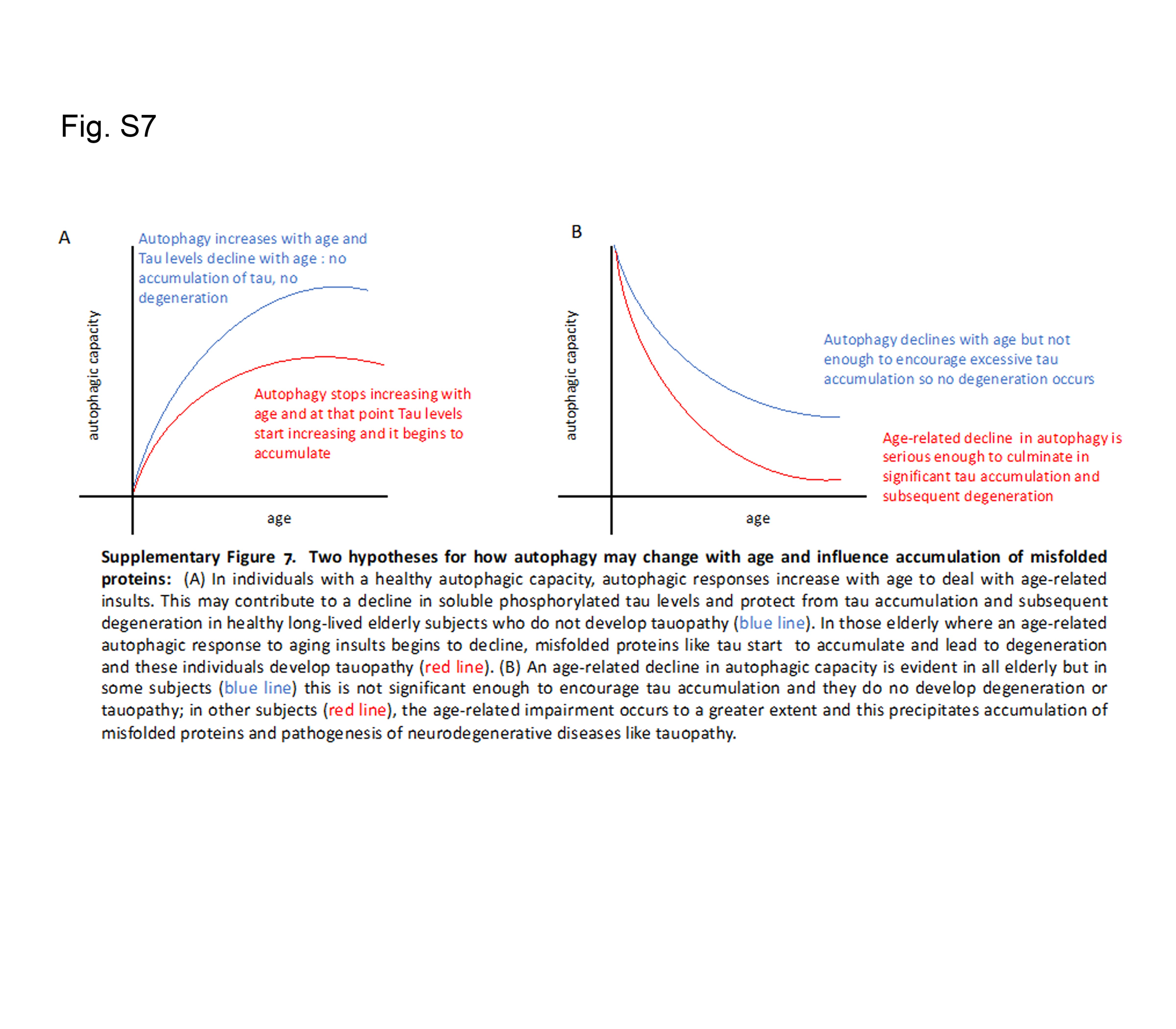

Supplement: S7 Fig — In individuals with a healthy autophagic capacity, autophagic responses increase with age to deal with age-related insults. This may contribute to a decline in soluble phosphorylated tau levels and protect from tau accumulation and sunsequent degeneration in healthy long-lived elderly subjects who do not develop tauopathy (red line) (A). An age-related decline in autophagic capacity is evident in all elderly but in some subjects (blue line) this is not significant enough to encourage tau accumulation and they do not develop degeneration or tauopathy in other subjects (red line), the age-related impairment occurs to a greater extent and this precipates accumulation of misfolded proteins and pathogenesis of neurodegenerative diseases like tauopathy. (TIF) [file pone.0262792.s007.tif]
