# Supplementary material for: Age-related changes in tau and autophagy in human brain in the absence of neurodegeneration
Source: PLoS One. 2023 Jan 26;18(1):e0262792. doi: 10.1371/journal.pone.0262792 (PMC9879510; doi:10.1371/journal.pone.0262792)

Set 1-Uncropped Blots for Tau and Actin in Resected and PM Tissue in Figure 1

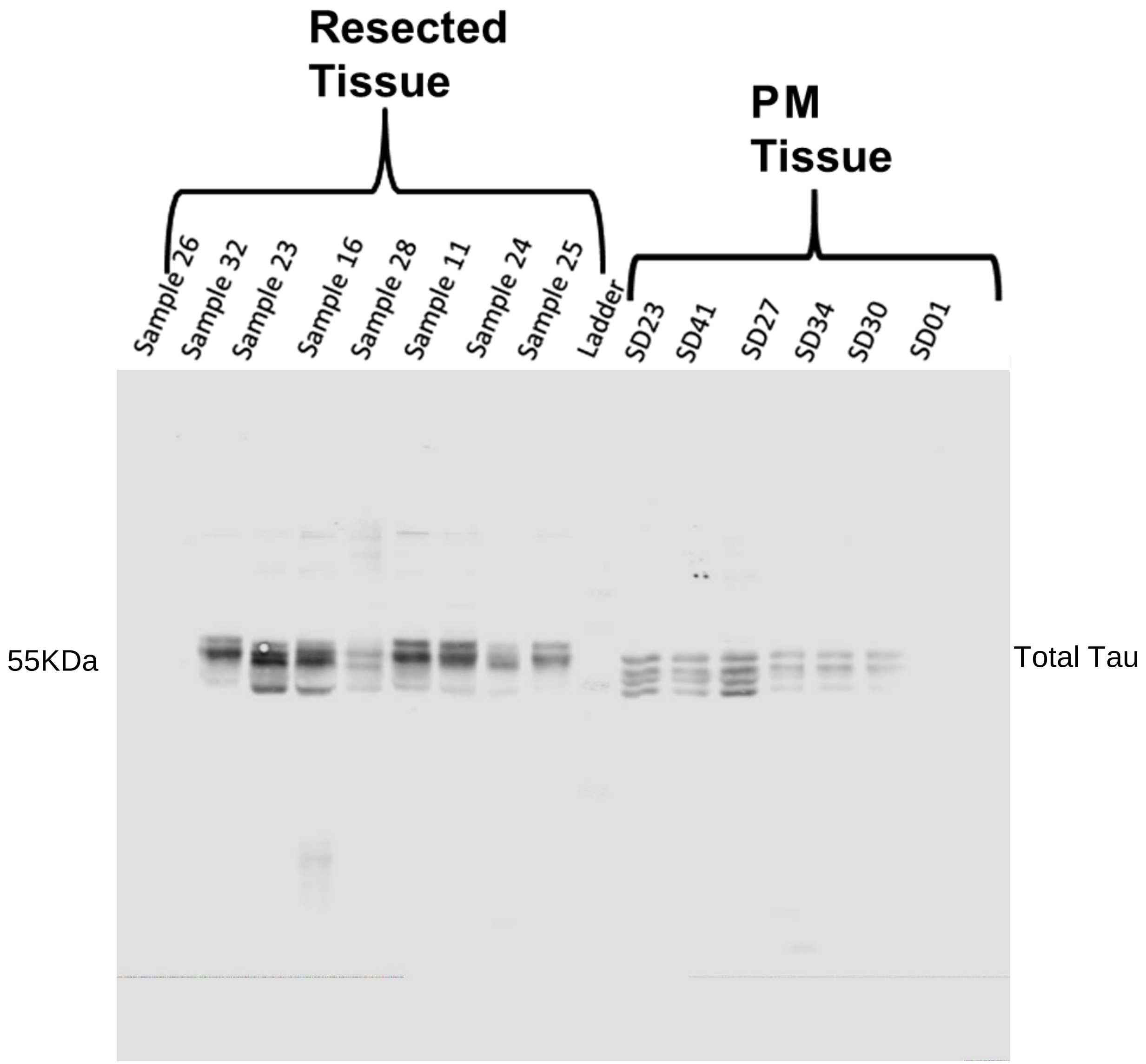

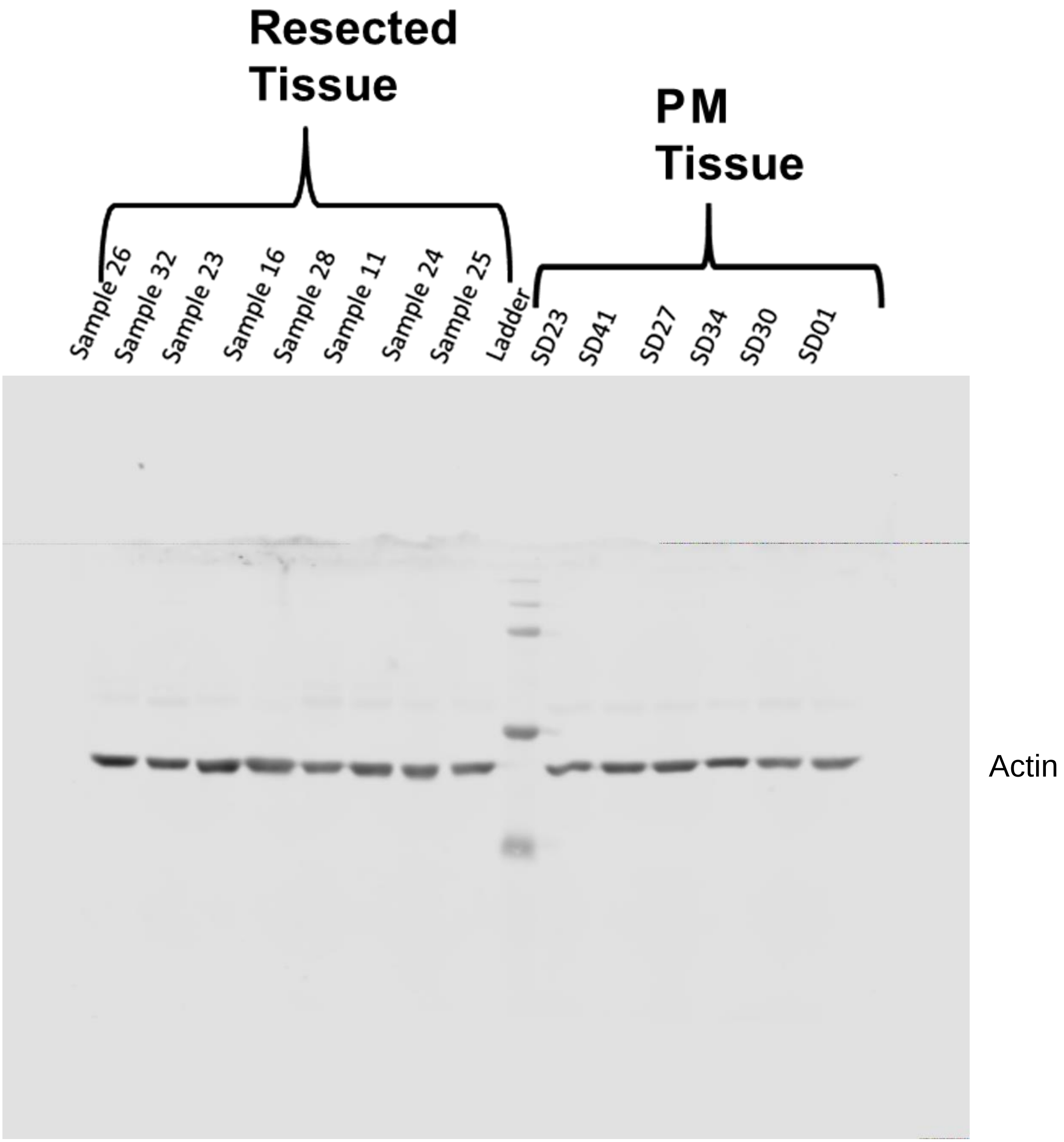

Set 1 – Uncropped Blots for PHF1 and Actin for Resected and PM Tissue in Fig.1

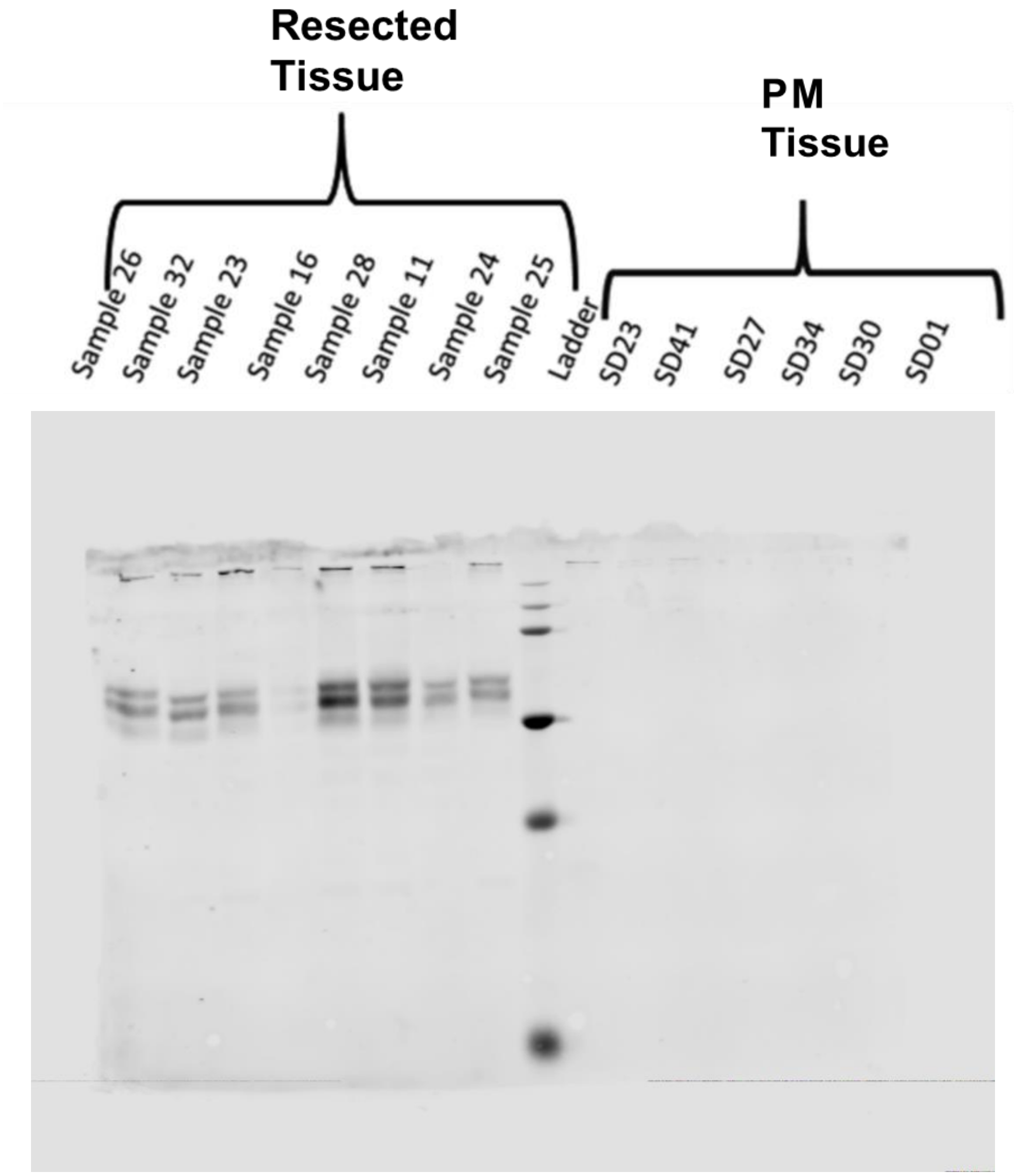

PHF1

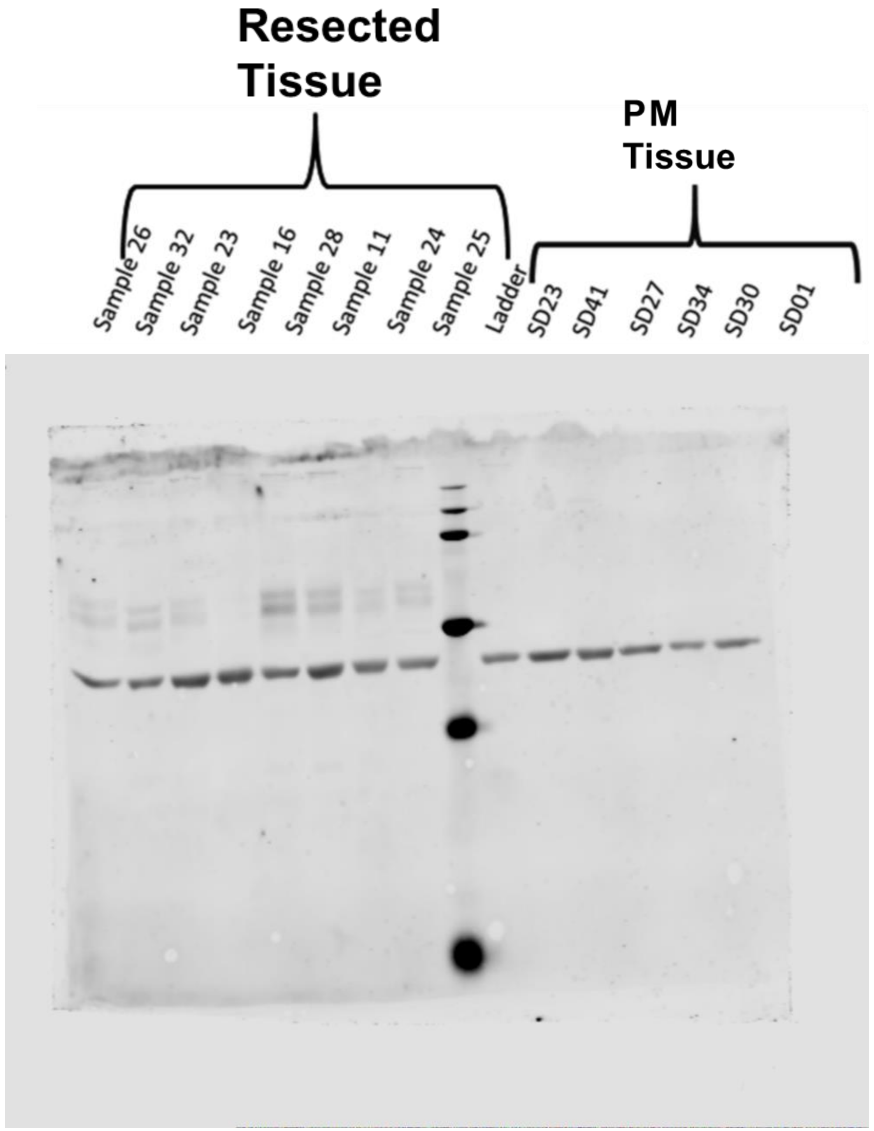

Actin

Set 2- Resected tissue Tau/PHF1 blots

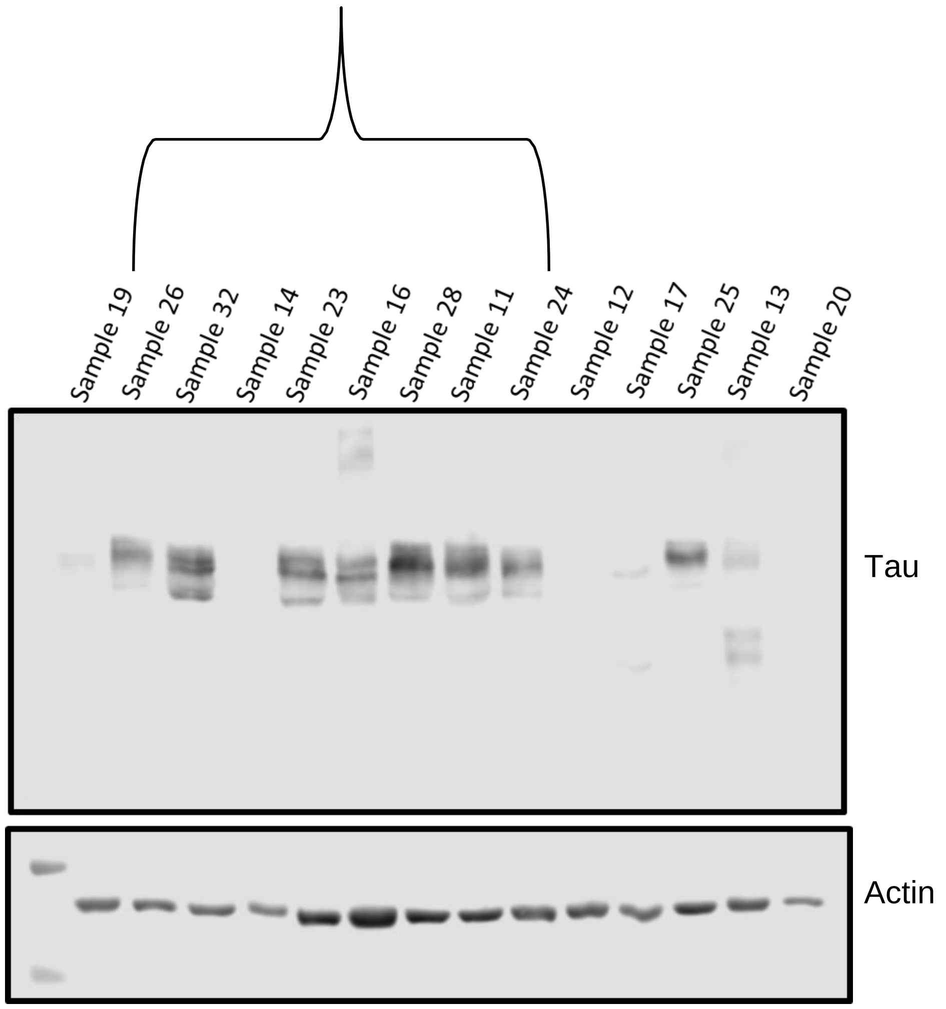

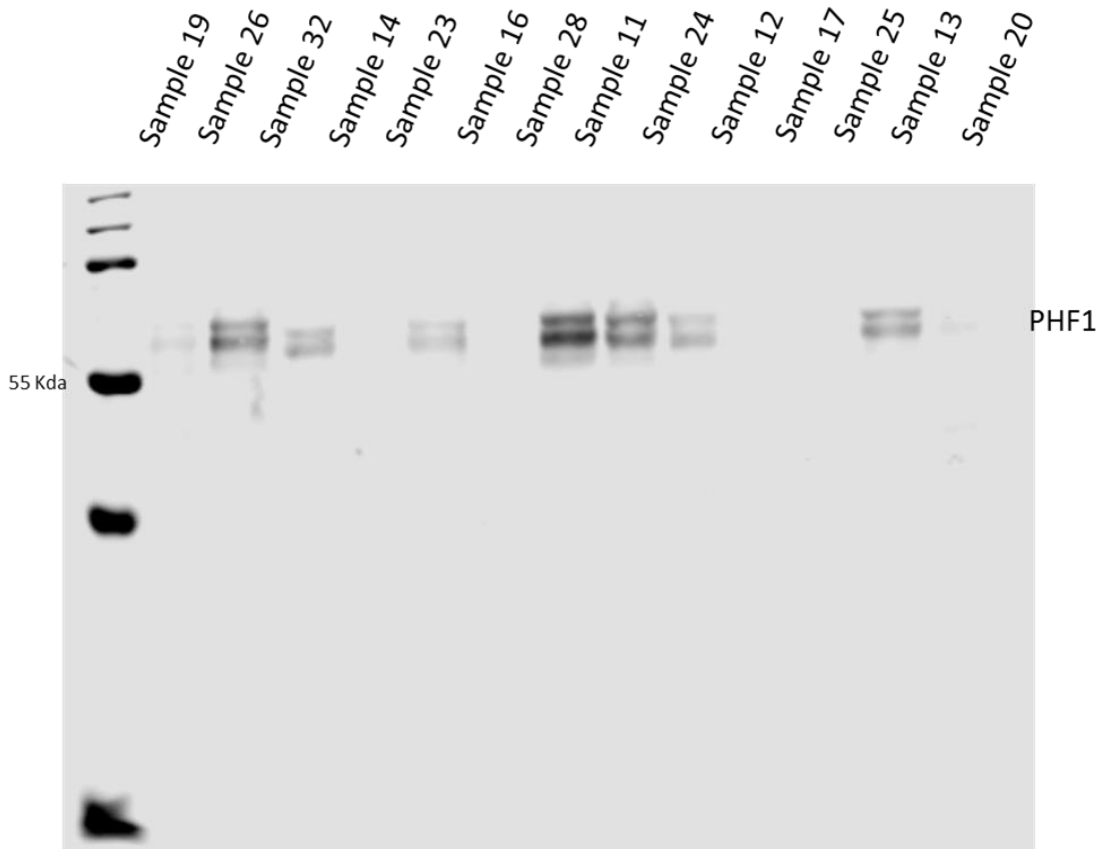

Set 2- Actin from Resected Tissue

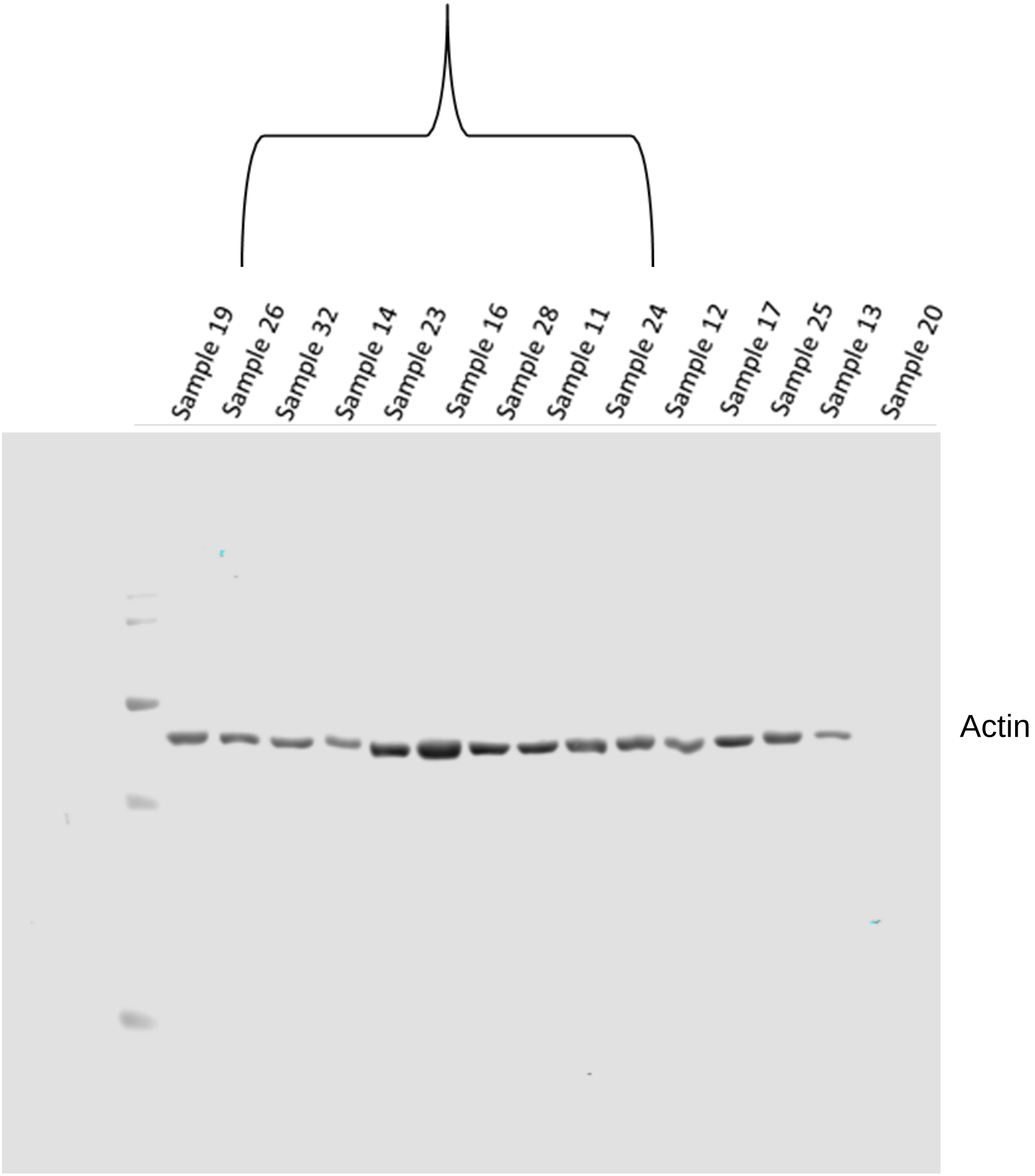

Set 3 –Resected tissue total Tau/PHF1 blots

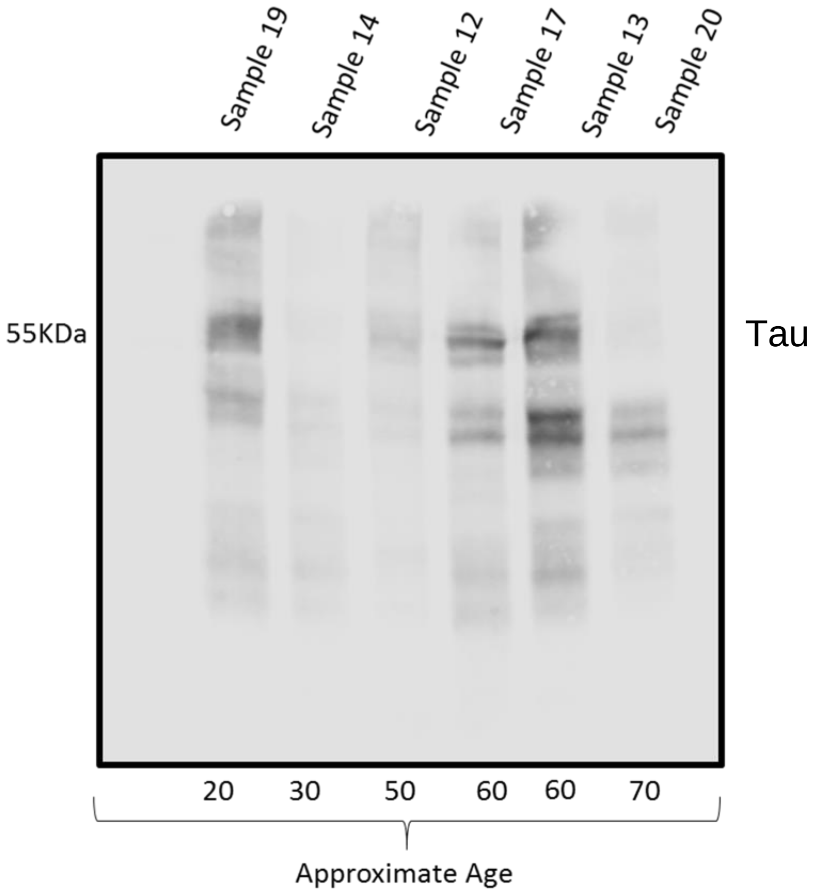

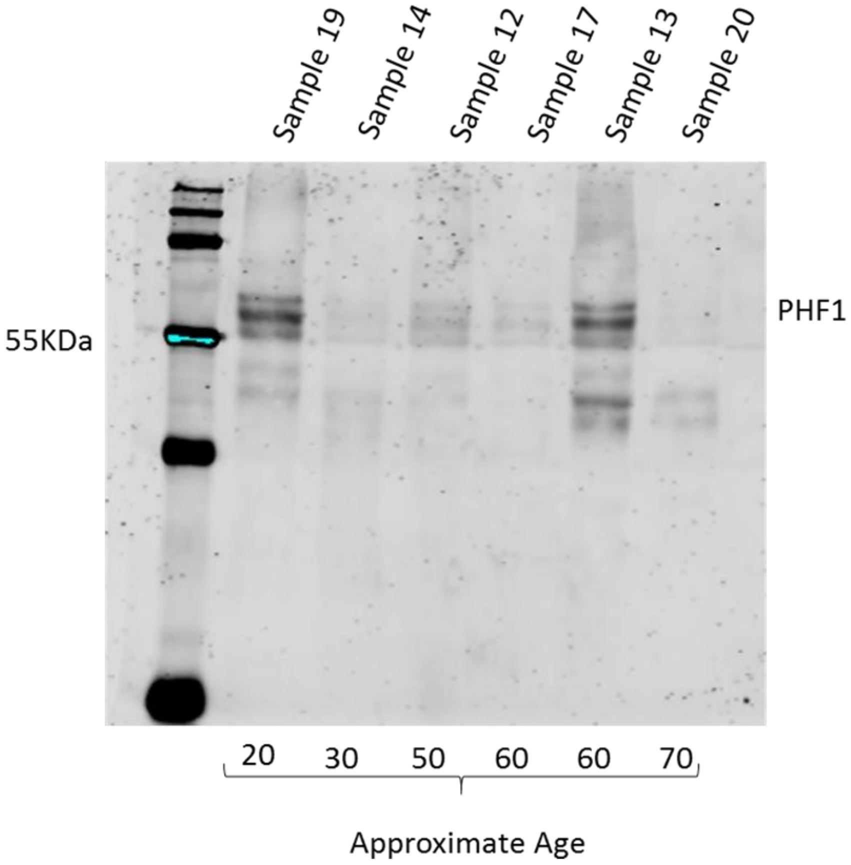

Set 2 and 3-PM tissue total Tau blots

22/2/18

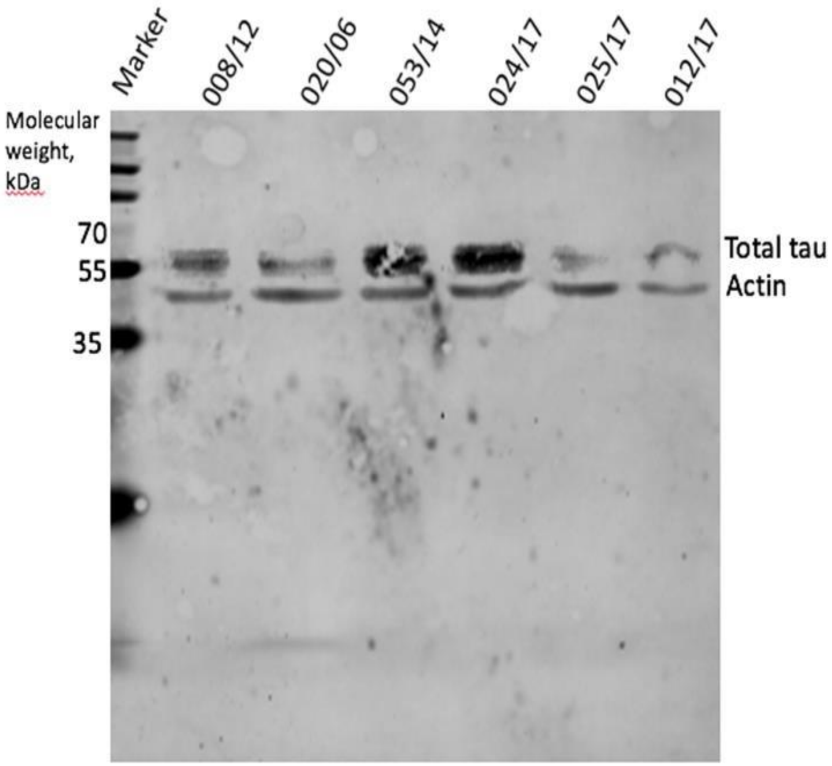

Actin MW = 42 kDa
Tau = kDa

19/4/18
Rb DAKO 1:10,000
Ms Actin 1:3,000
Gel A: Anti-rb and anti-ms 1:20,000
Gel B: Anti-rb and anti-ms 1:15,000

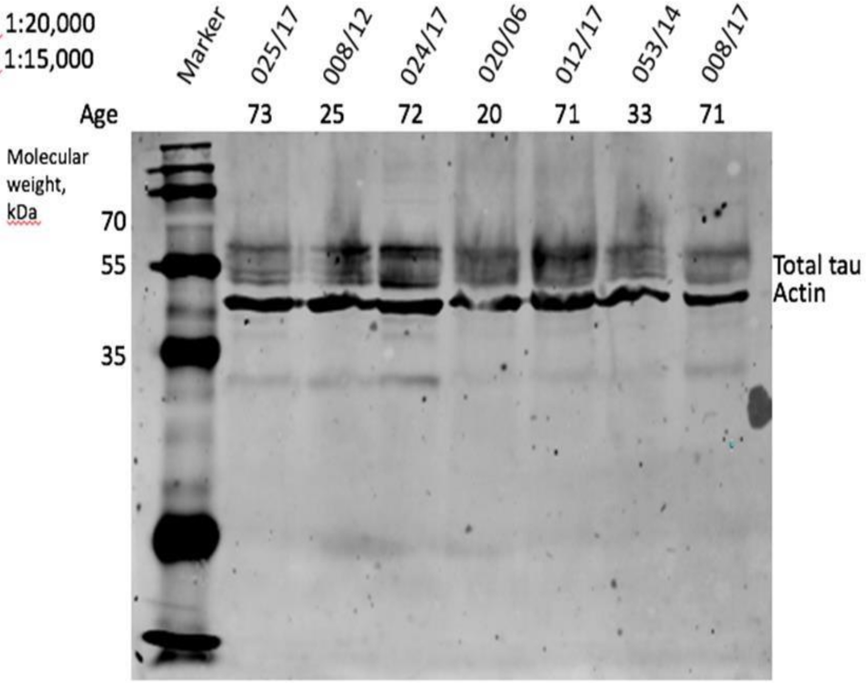

Actin = 42 kDa
Tau =

Set 1- Uncropped Blots for Acetylated-Tubulin and GAPDH for PM tissue Fig. 2A

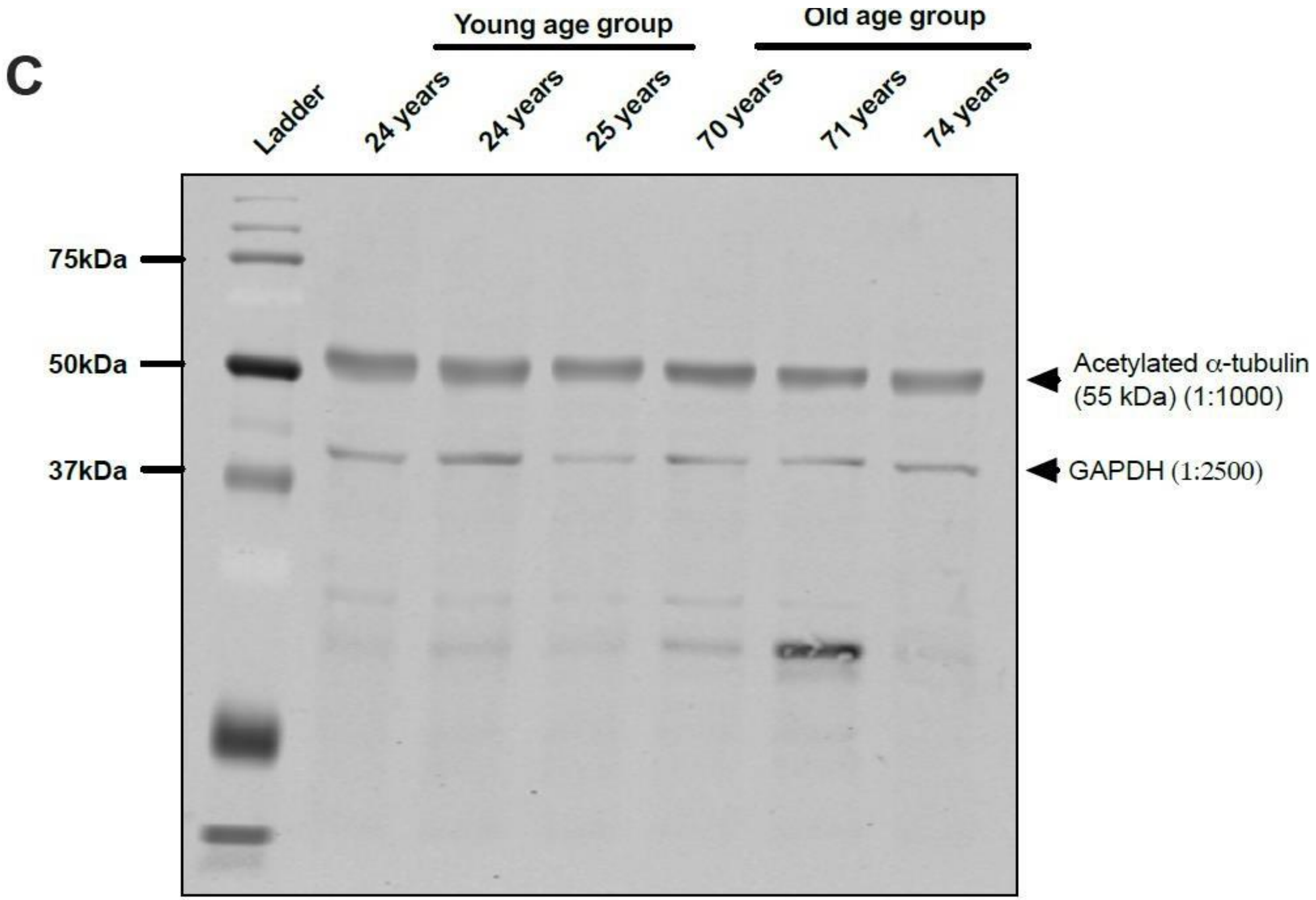

This blot is part of Figure 2 A

Set 2 and 3 – PM tissue Acetylated α-tubulin

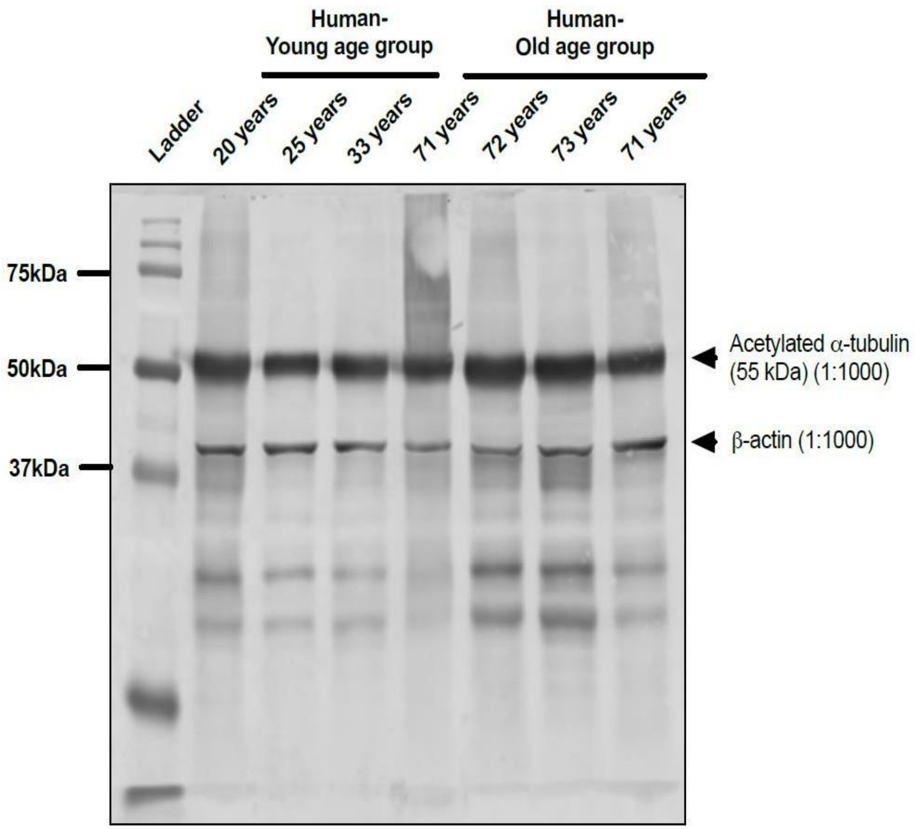

C

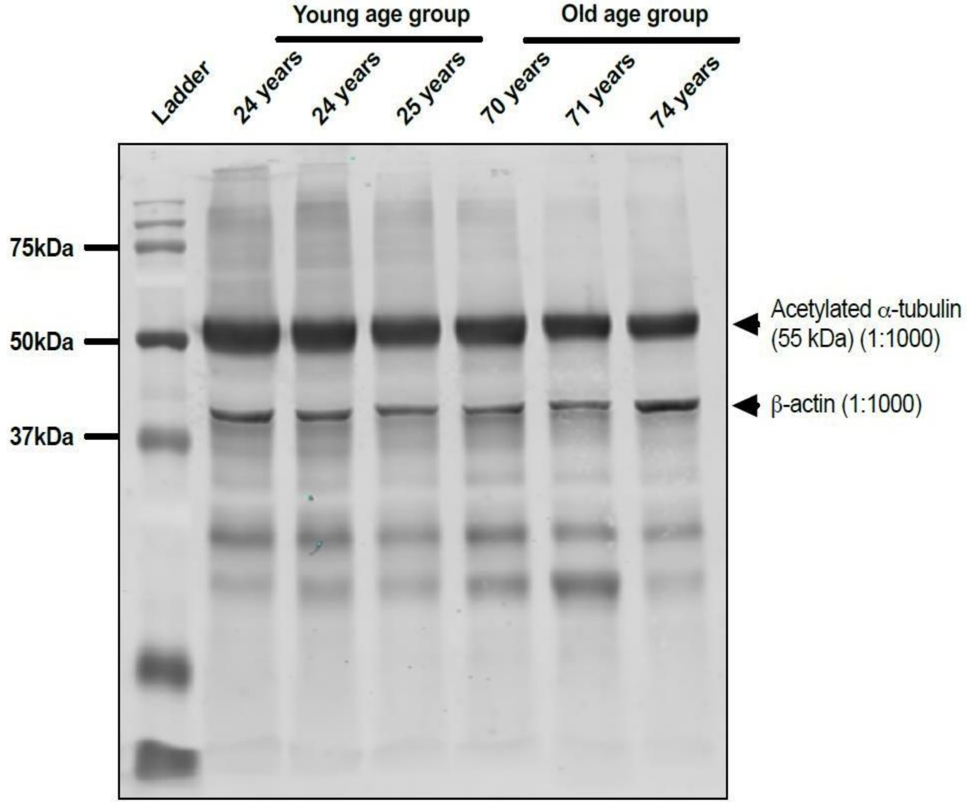

Set 1- Uncropped blot for Tyrosinated Tubulin and GAPDH for PM tissue in Fig. 2B

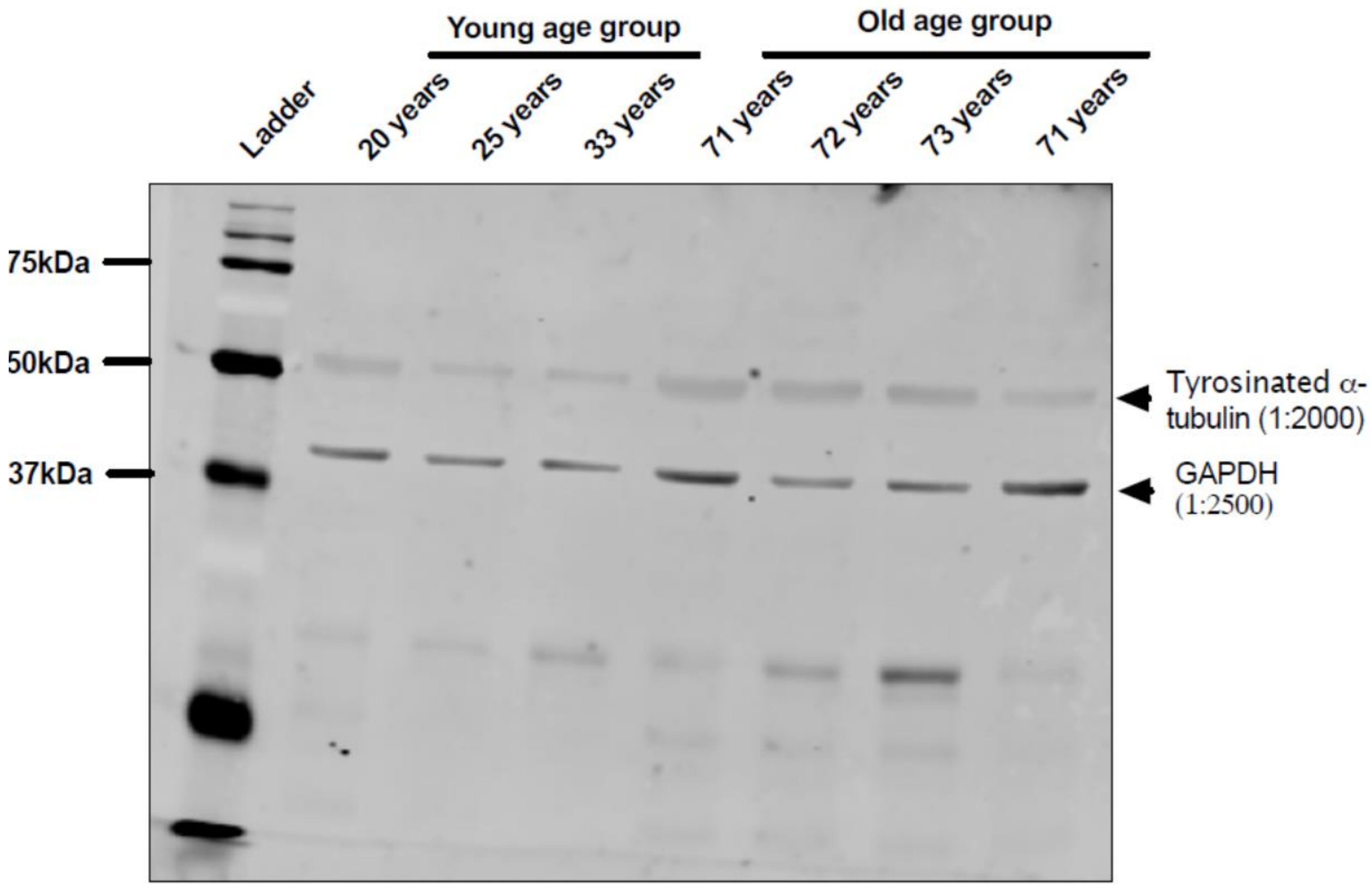

This blot is part of Figure 2 B

Set 2 and 3- PM tissueTyrosinated tubulin and GAPDH

Repeat 1

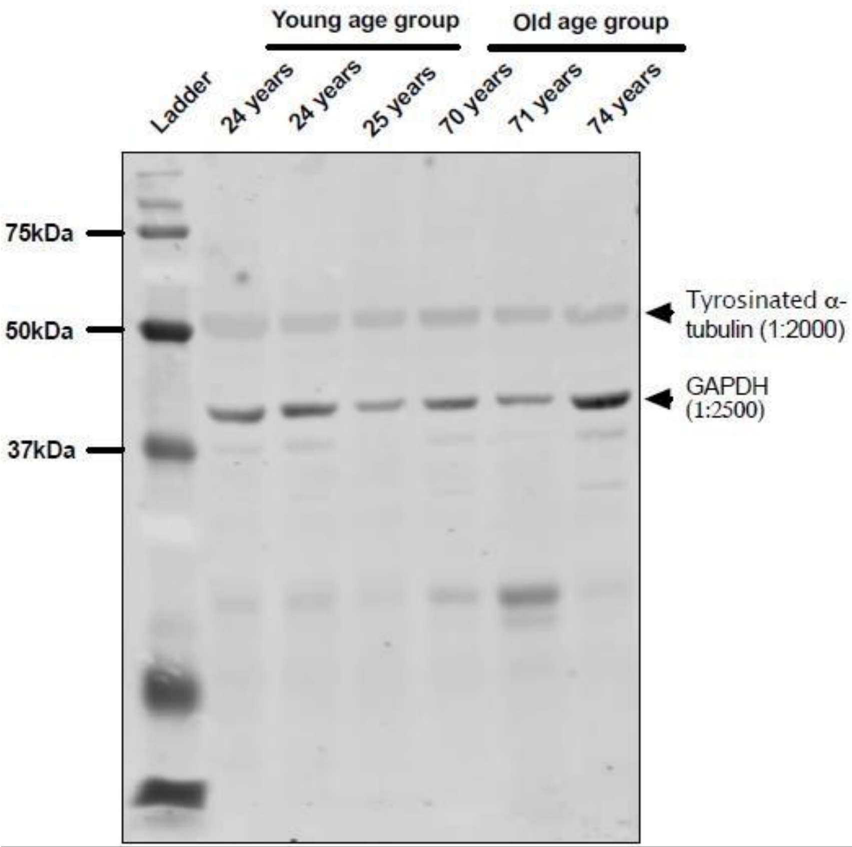

Repeat 2

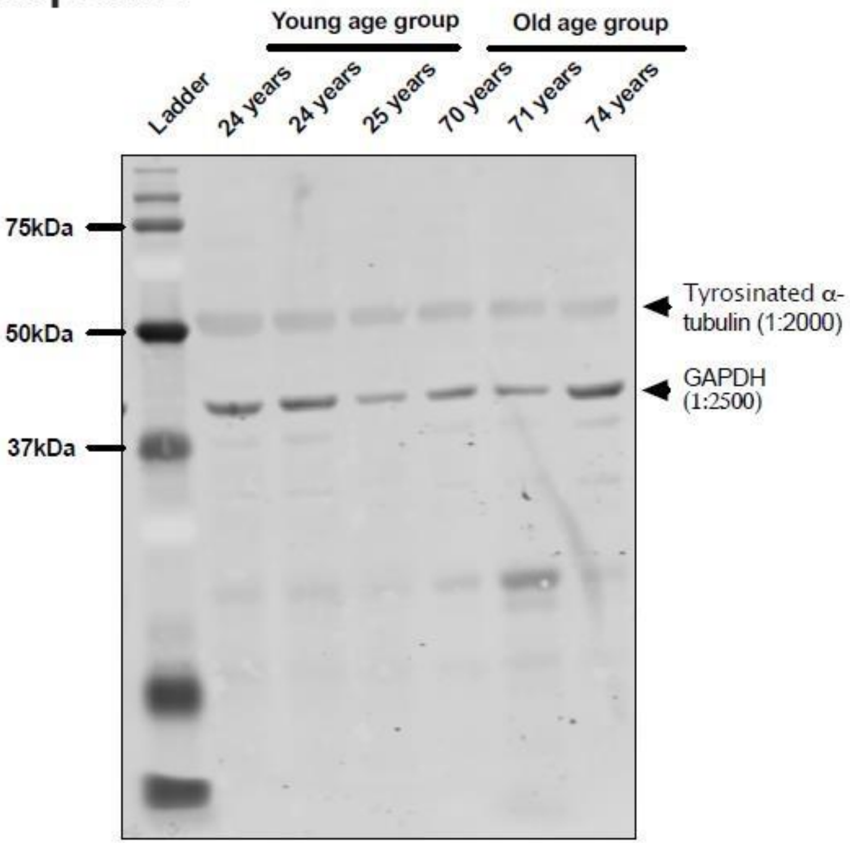

Set 1 : Uncropped blots of p62 and Actin in PM tissues in Fig. 3A

28/2/18

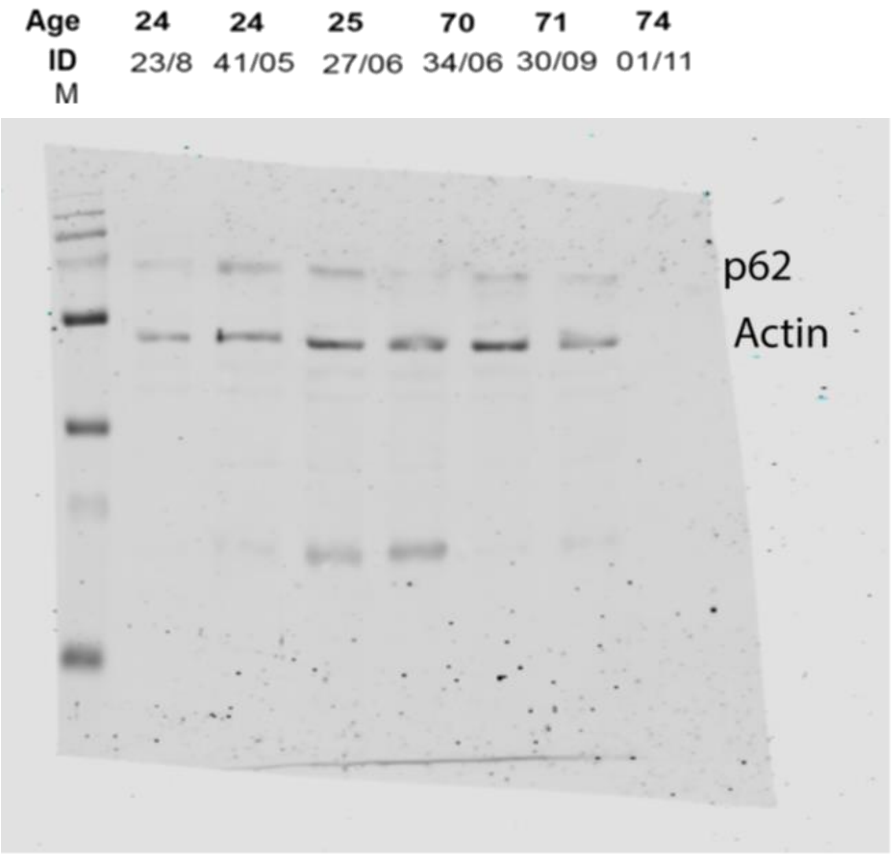

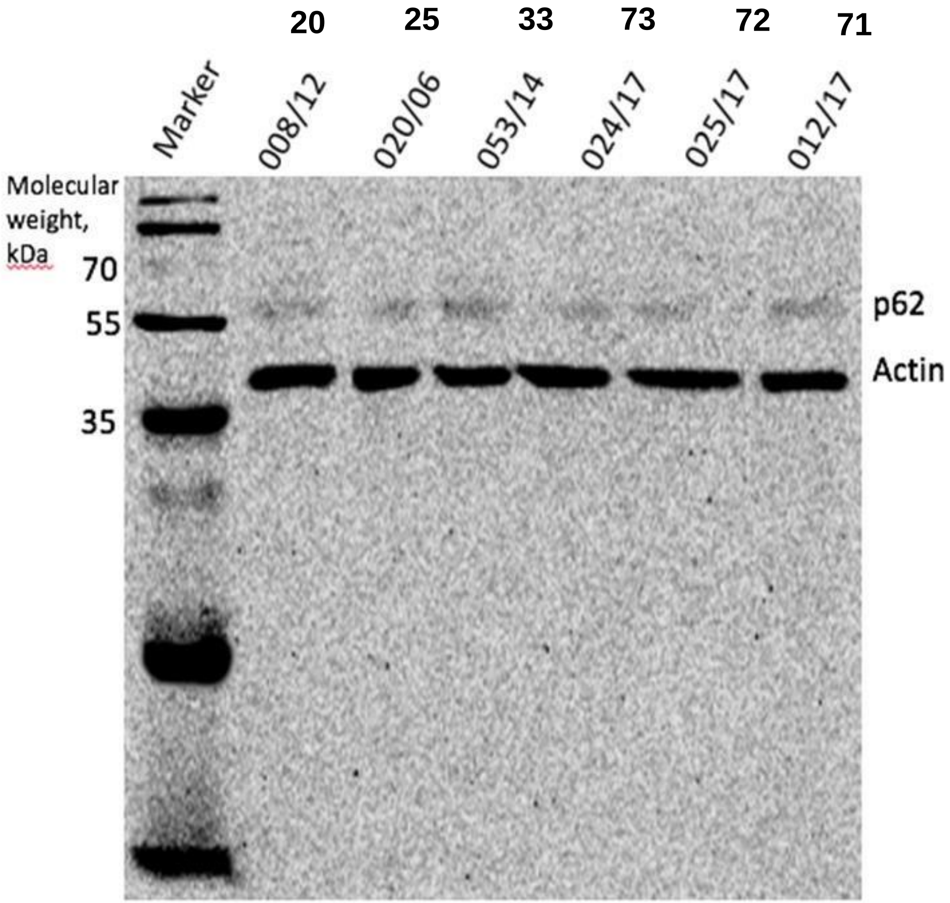

Actin MW = 42 kDa
p62 = 62 kDa

Set 2: Uncropped blots of p62 and Actin in PM Tissue

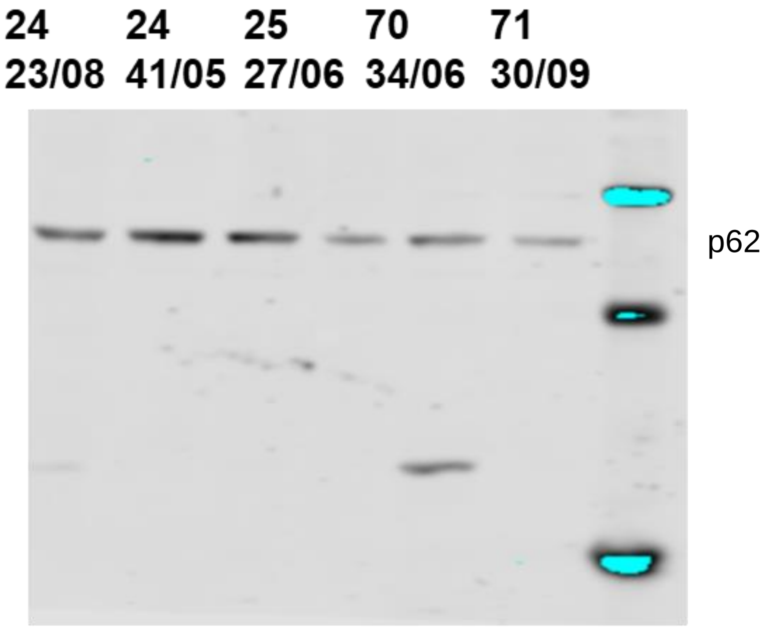

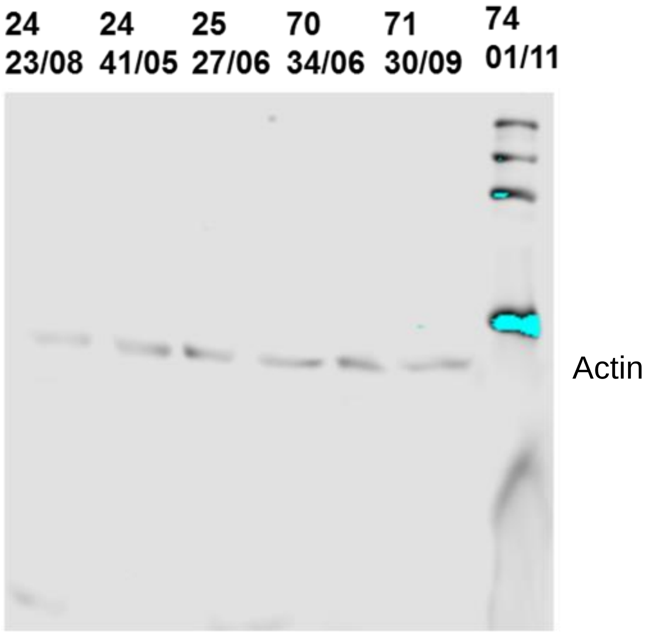

28/2/18

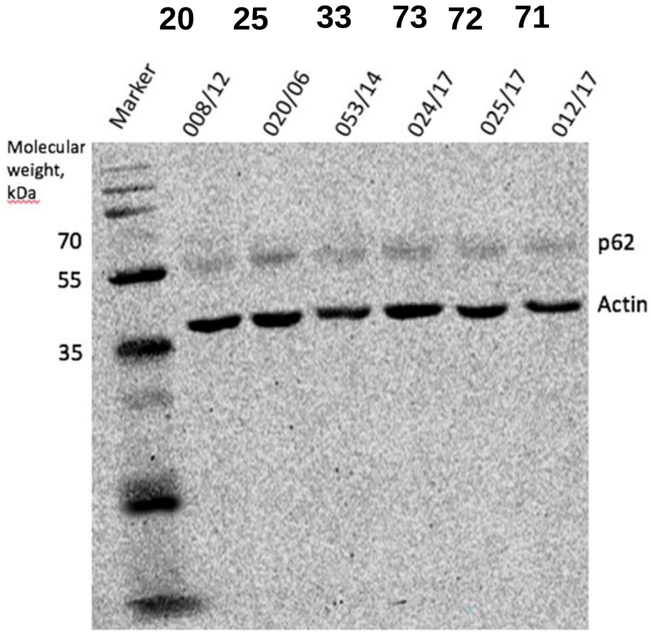

Actin MW = 42 kDa
p62 = 62 kDa

Set 3-Uncropped Blots pf p62 and Actin in PM tissues

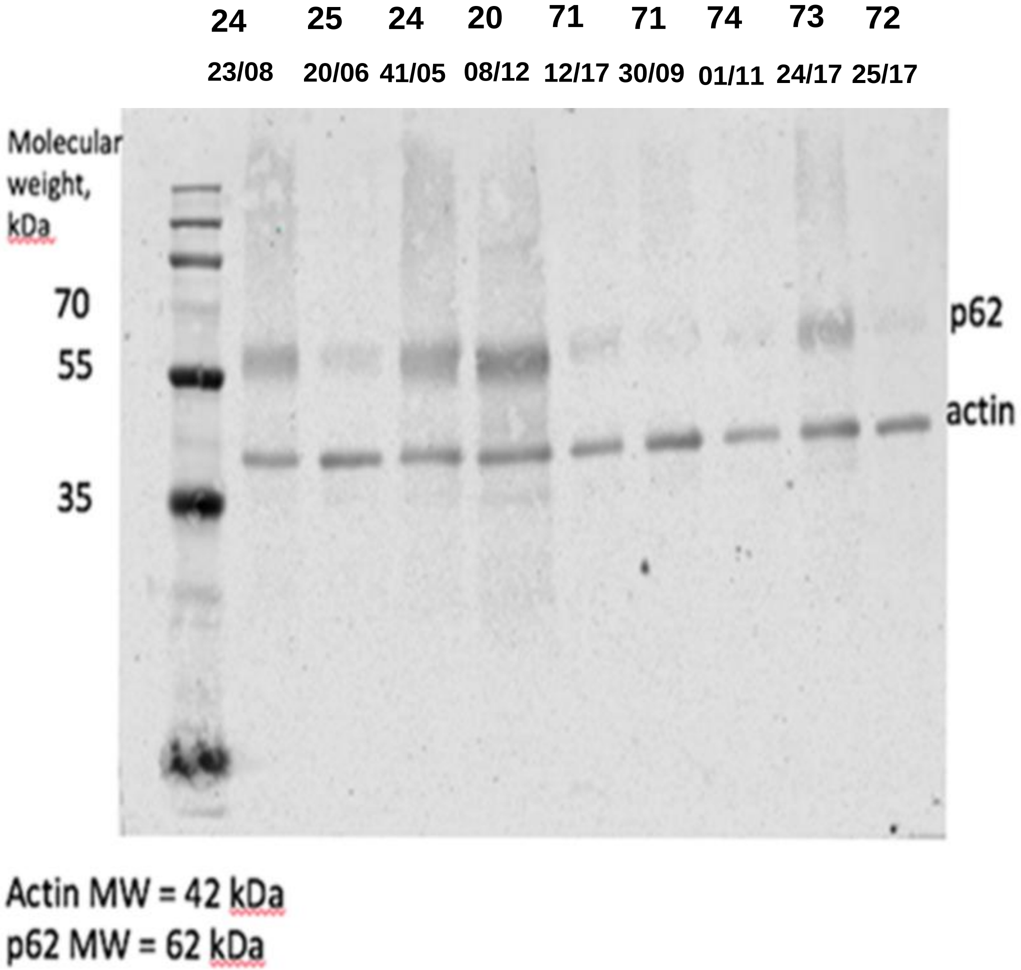

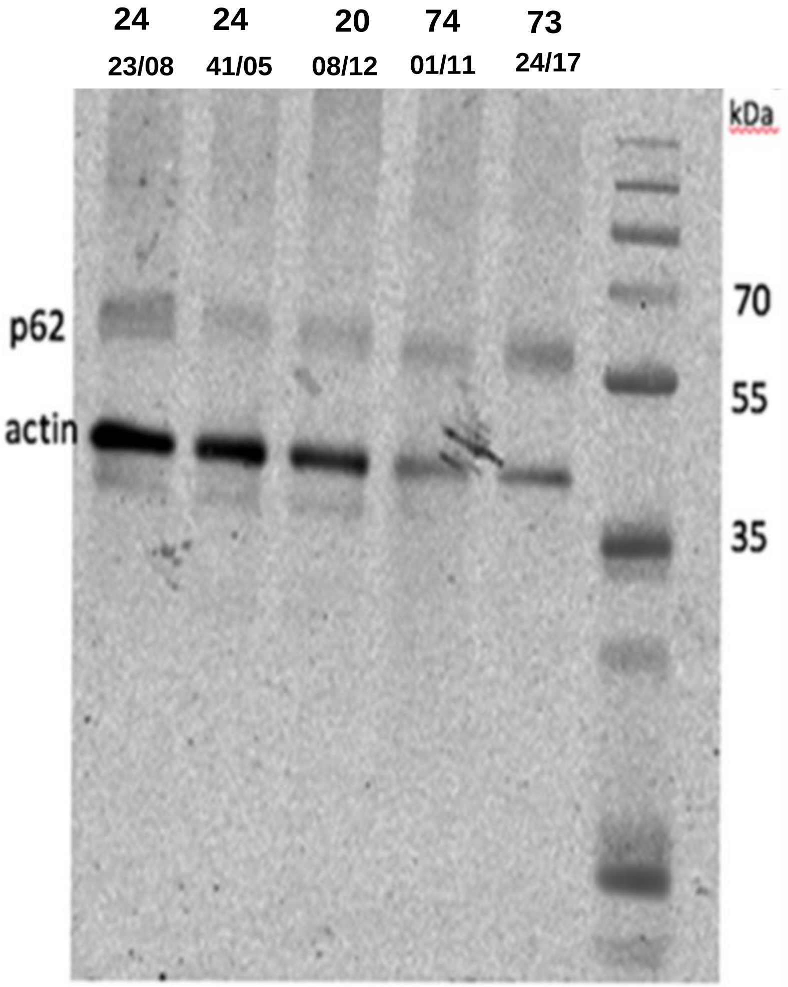

Set 1- Uncropped blot for LC3 and Actin for Fig.3B in PM tissues

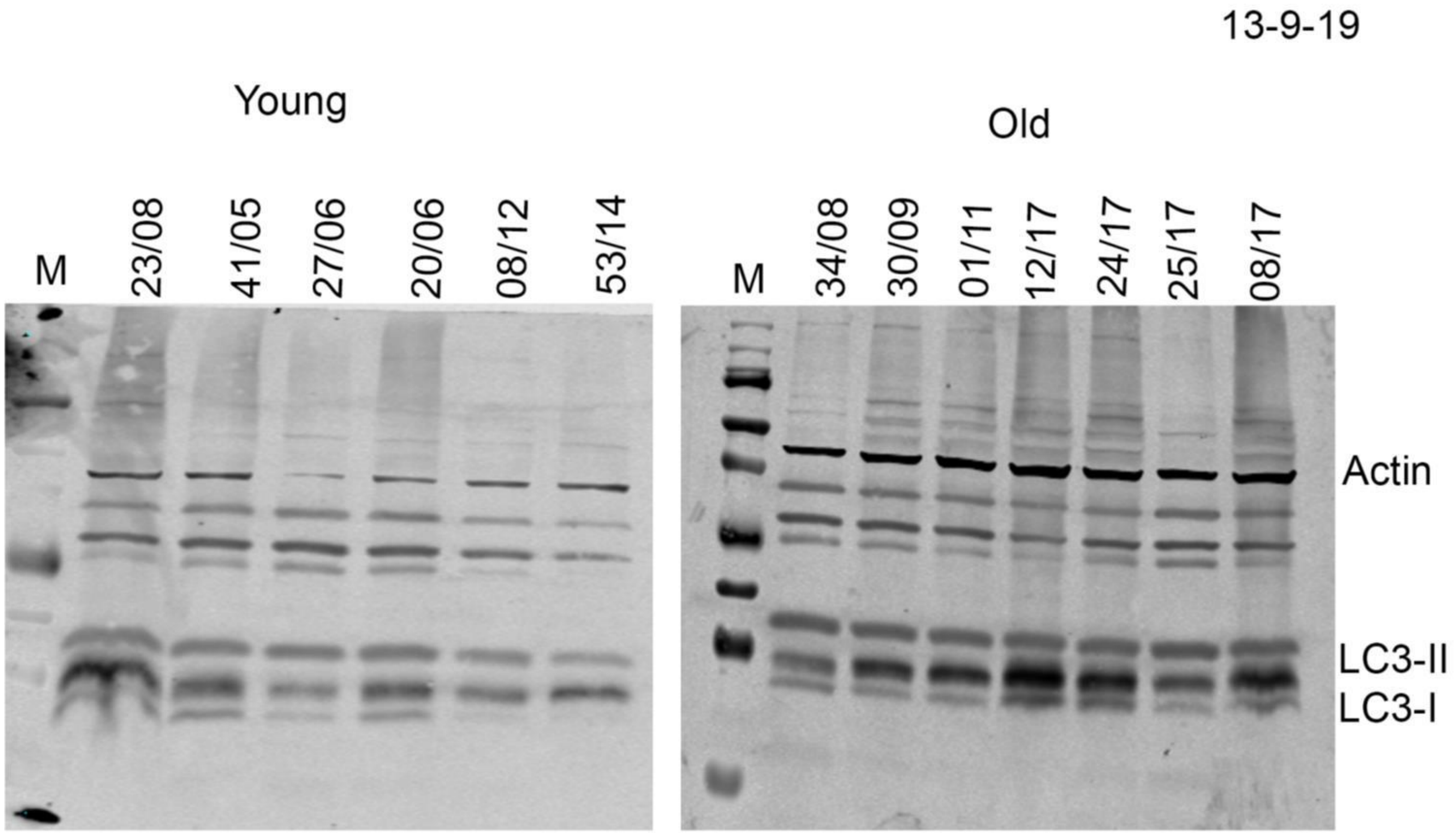

Young

23/08 – 24 years
41/05 – 24 years
27/06 – 25 years
20/06 – 25 years
08/12 – 20 years
53/14 – 33 years

Old

34/08 - 70 years
30/09 – 71 years
01/11 – 74 years
12/17 – 73 years
24/17 – 72 years
25/17 – 71 years
08/17 – 71 years

Set 2 LC3 and Actin in PM tissues

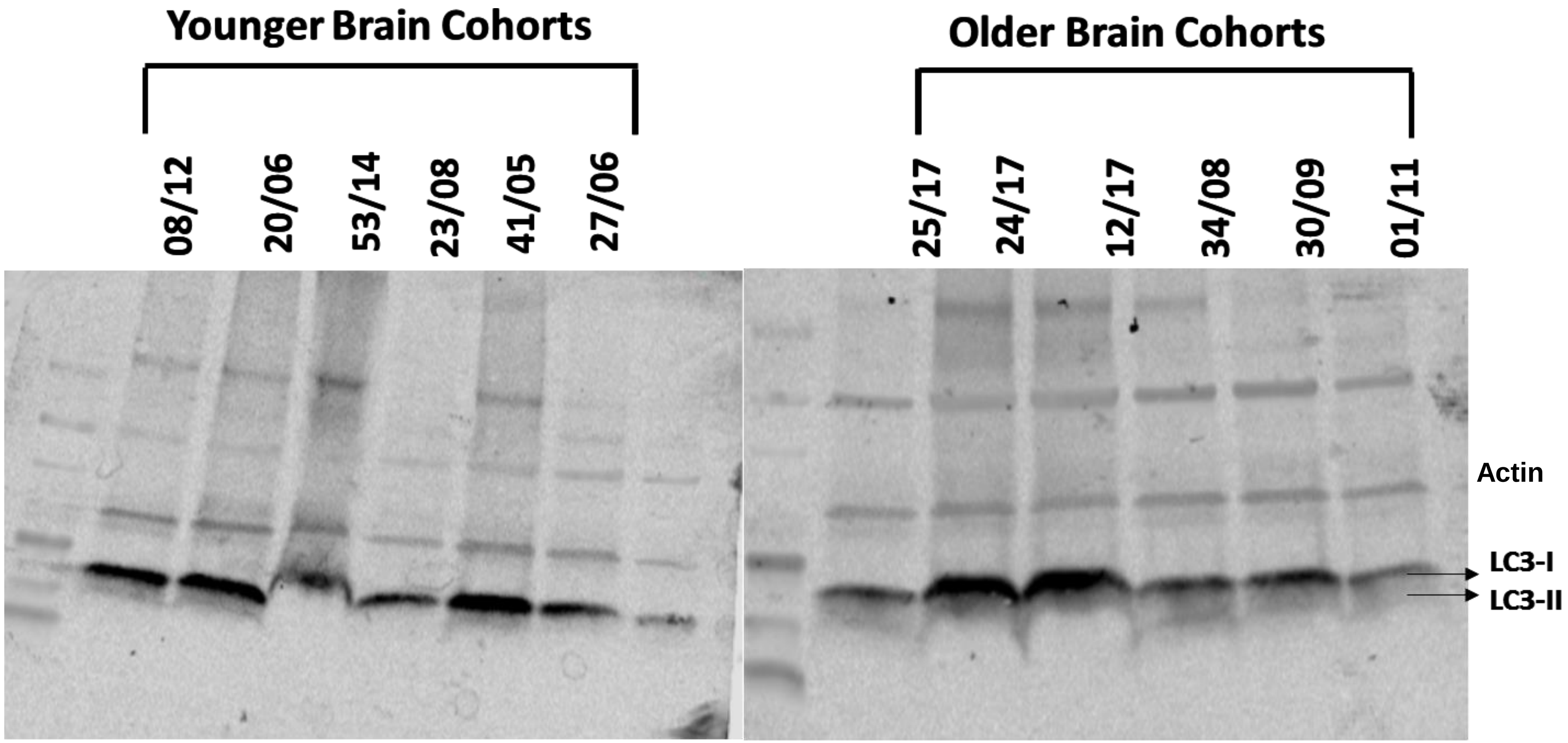

Set1 – Uncropped Beclin 1 and Actin in PM tissues in Fig.4

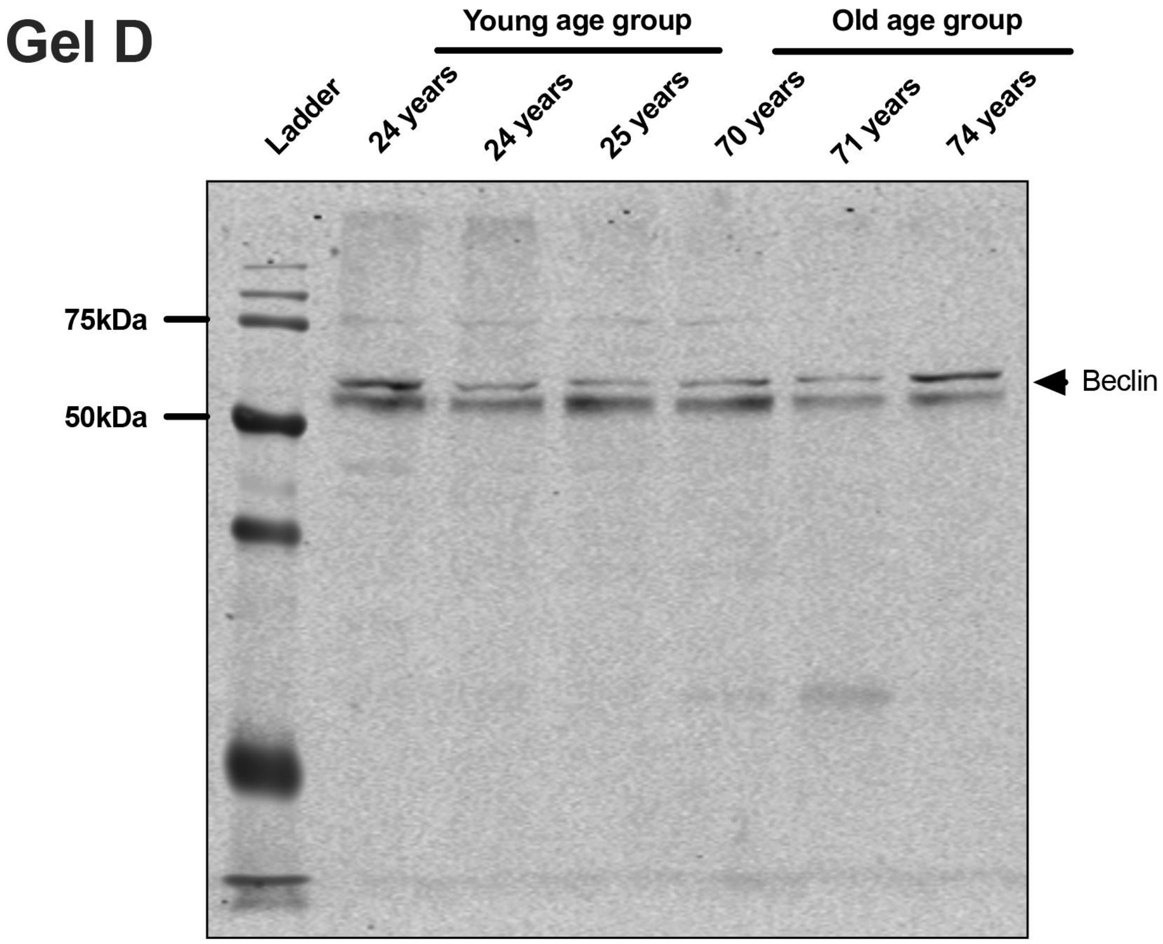

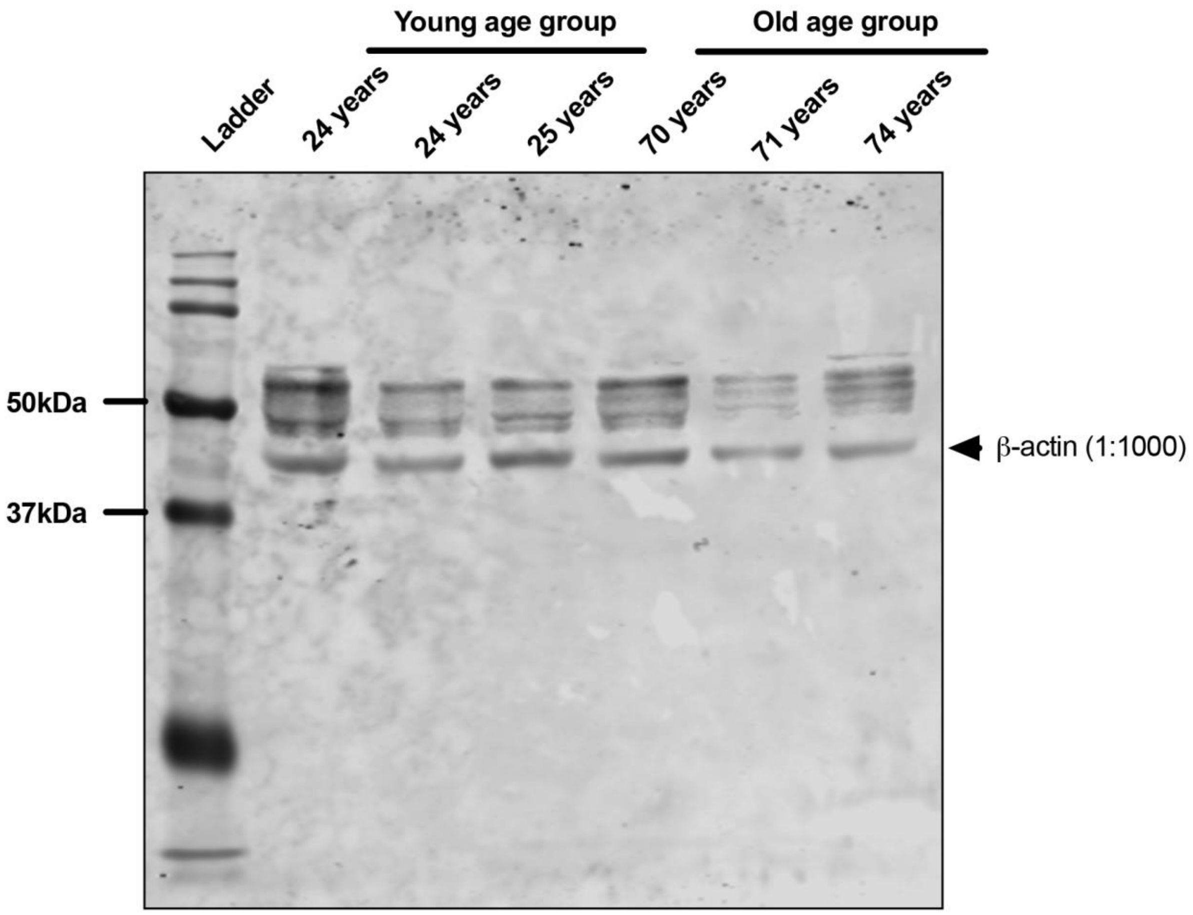

Set 2 and 3 – Beclin 1 and Actin blots for PM tissues

Gel C

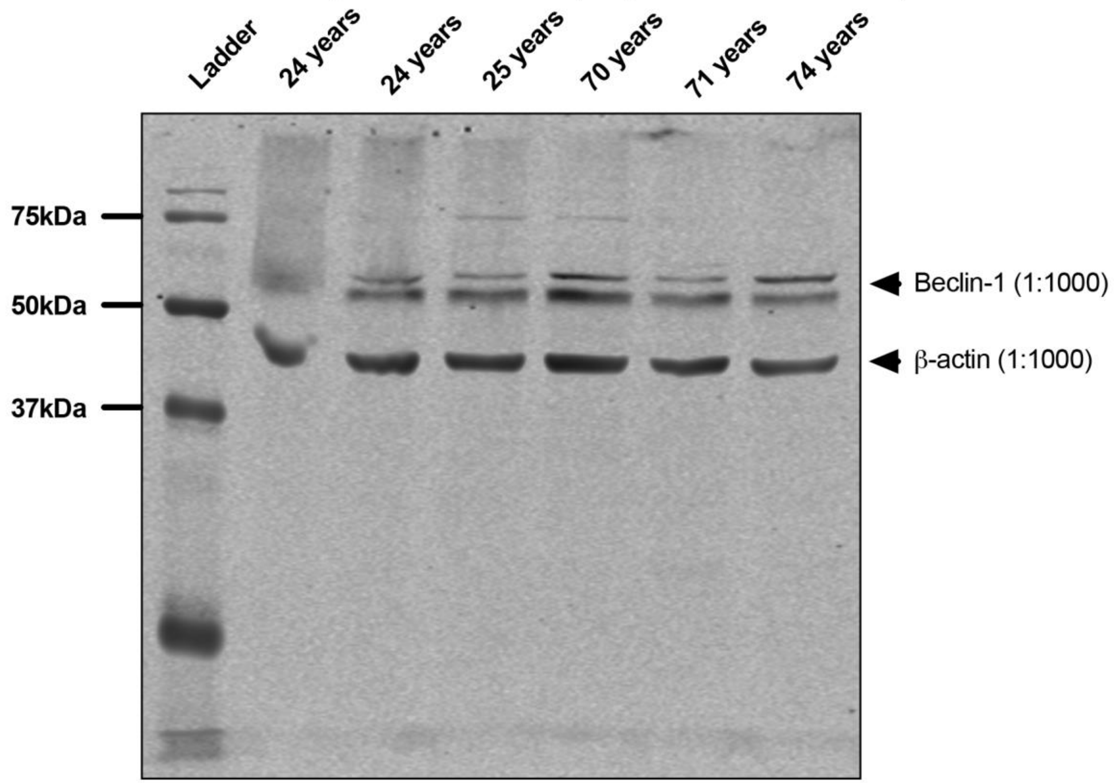

.8 Gel B

lin-1 1:1,000
in 1:3,000
and anti-ms 1:20,000

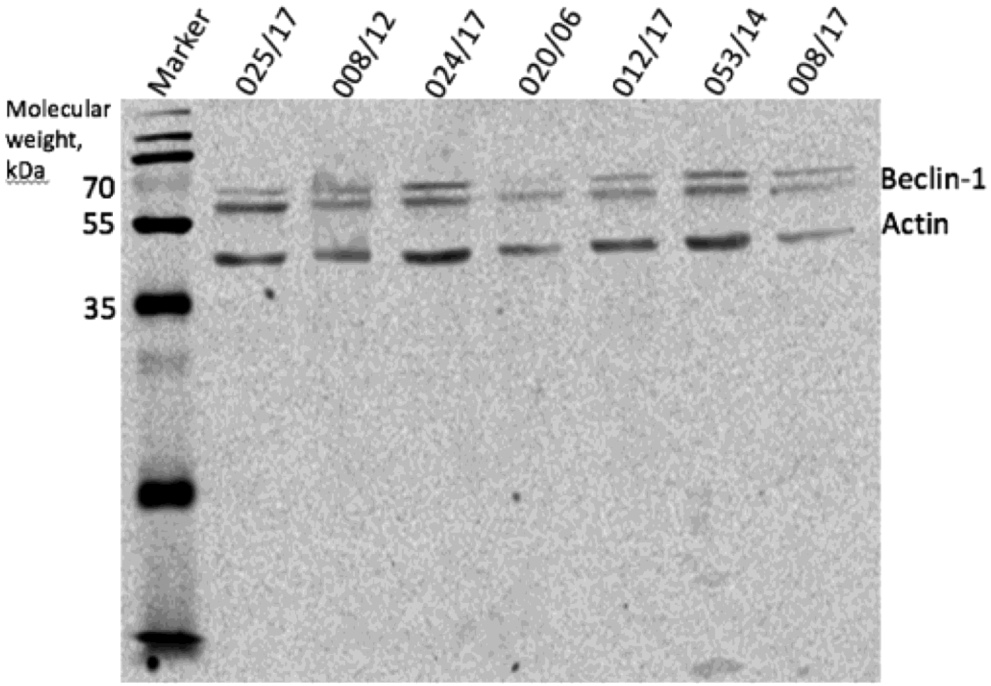

: 42 kDa
Beclin-1 = 52 kDa

Set 1 and 2 – Uncropped blots for human Tau and Actin for Drosophila lines in Fig. 5A

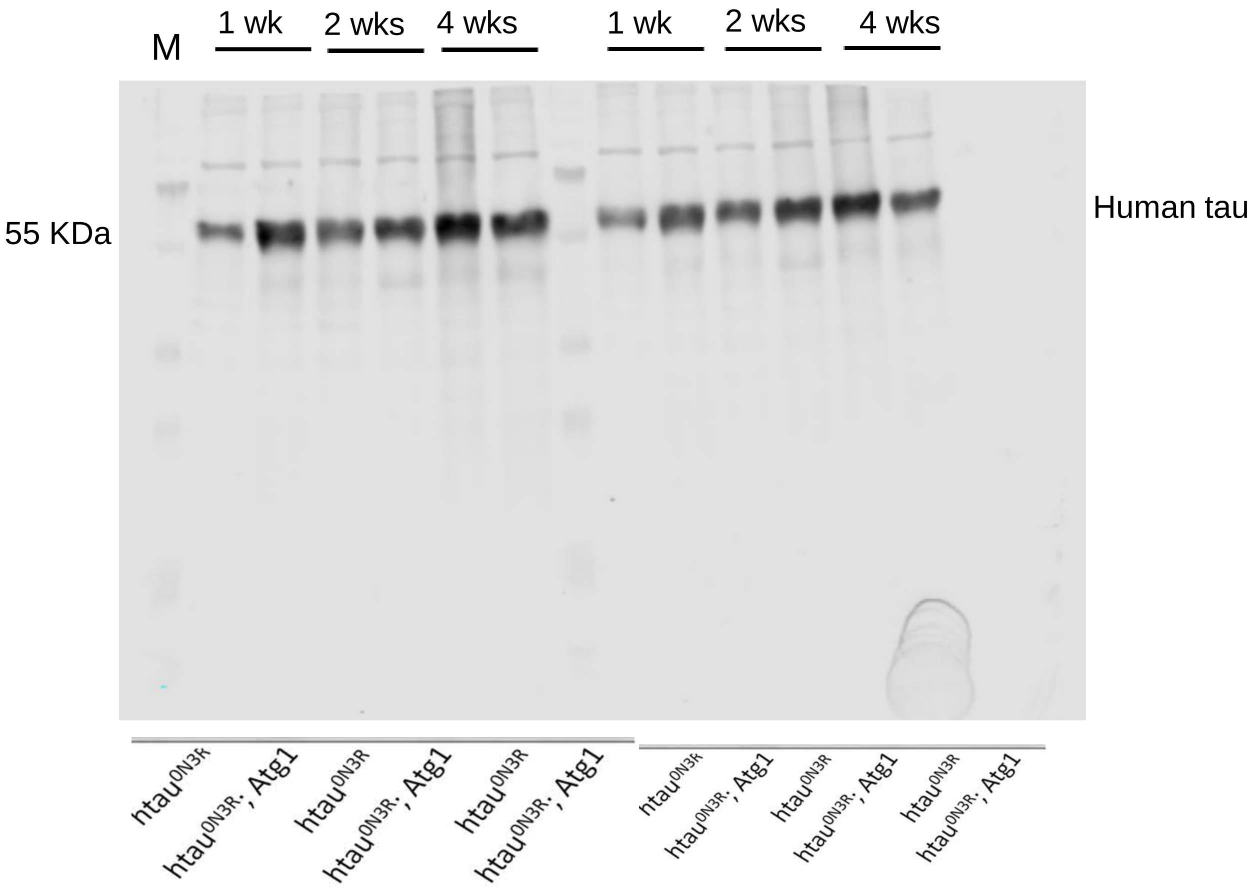

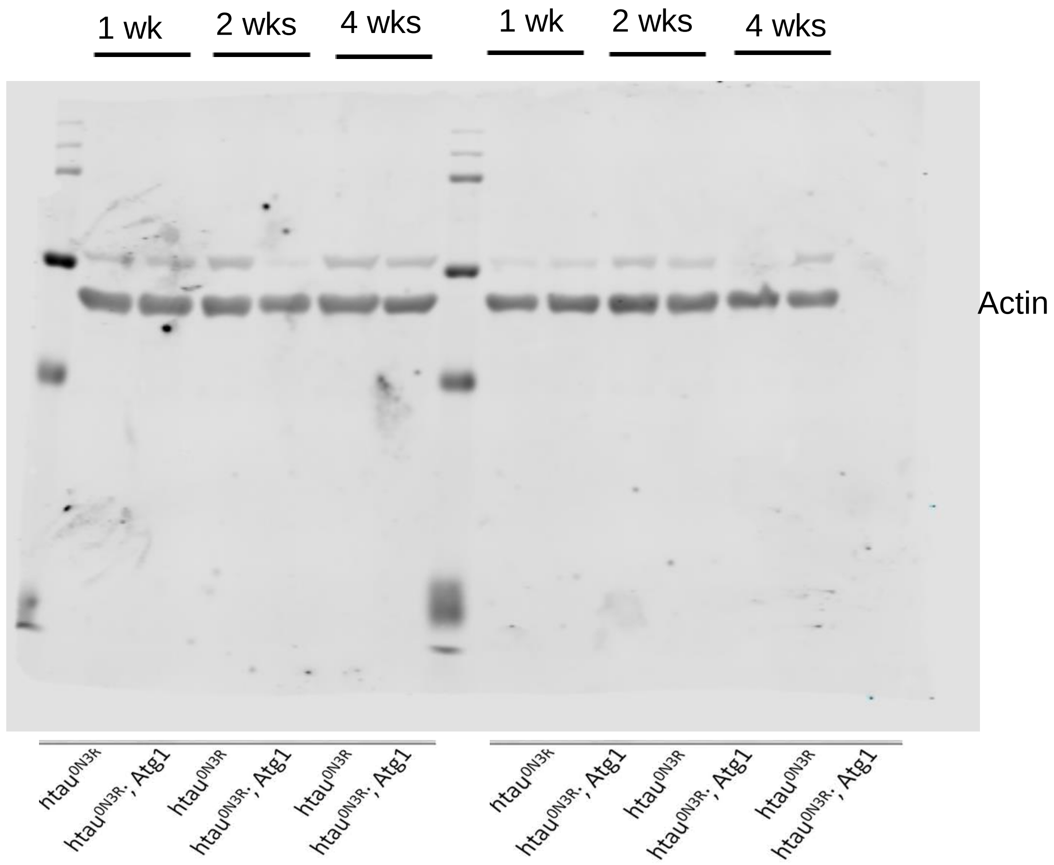

Set 1 and 2 – Uncropped blots for PHF1 and Actin for Drosophila lines in Fig. 5A

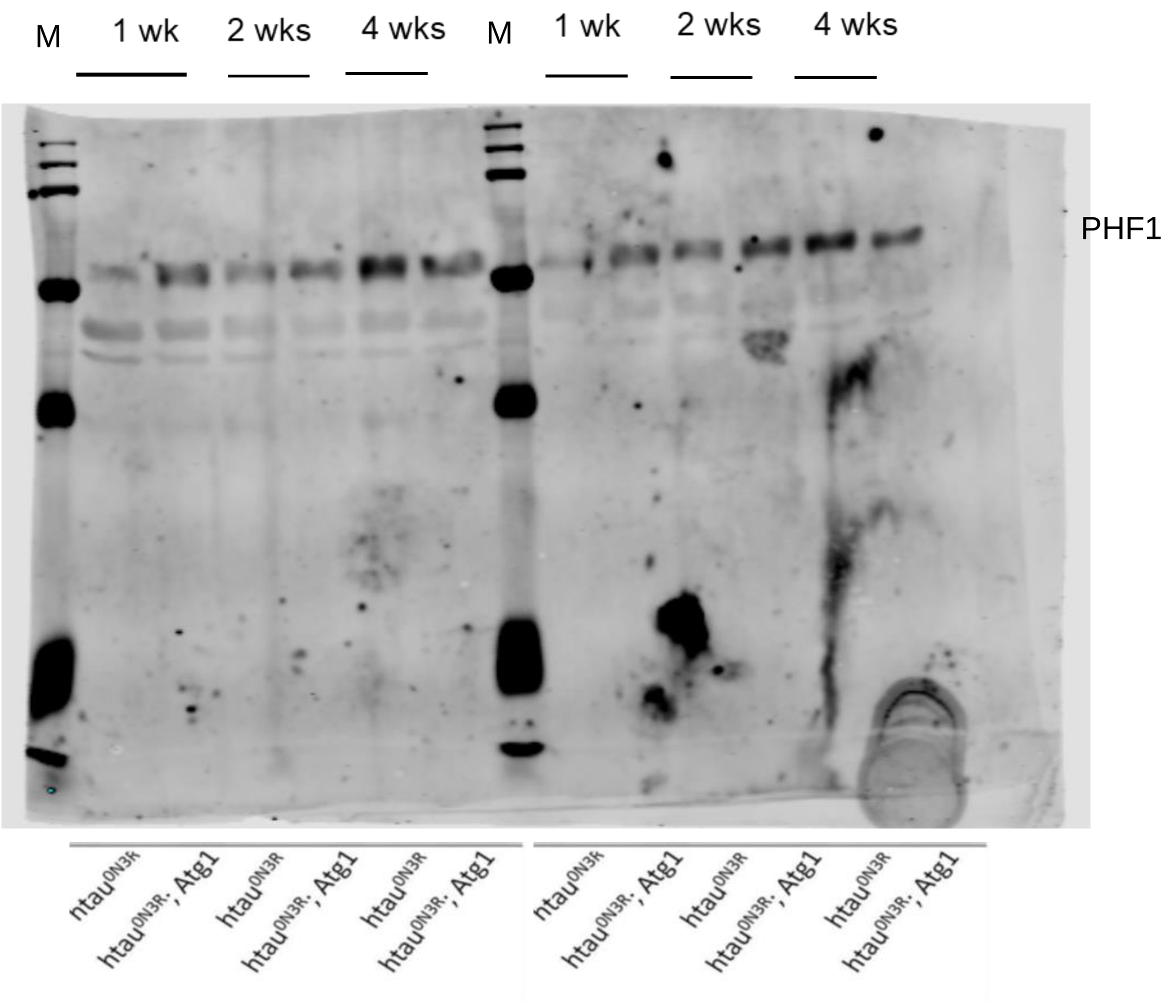

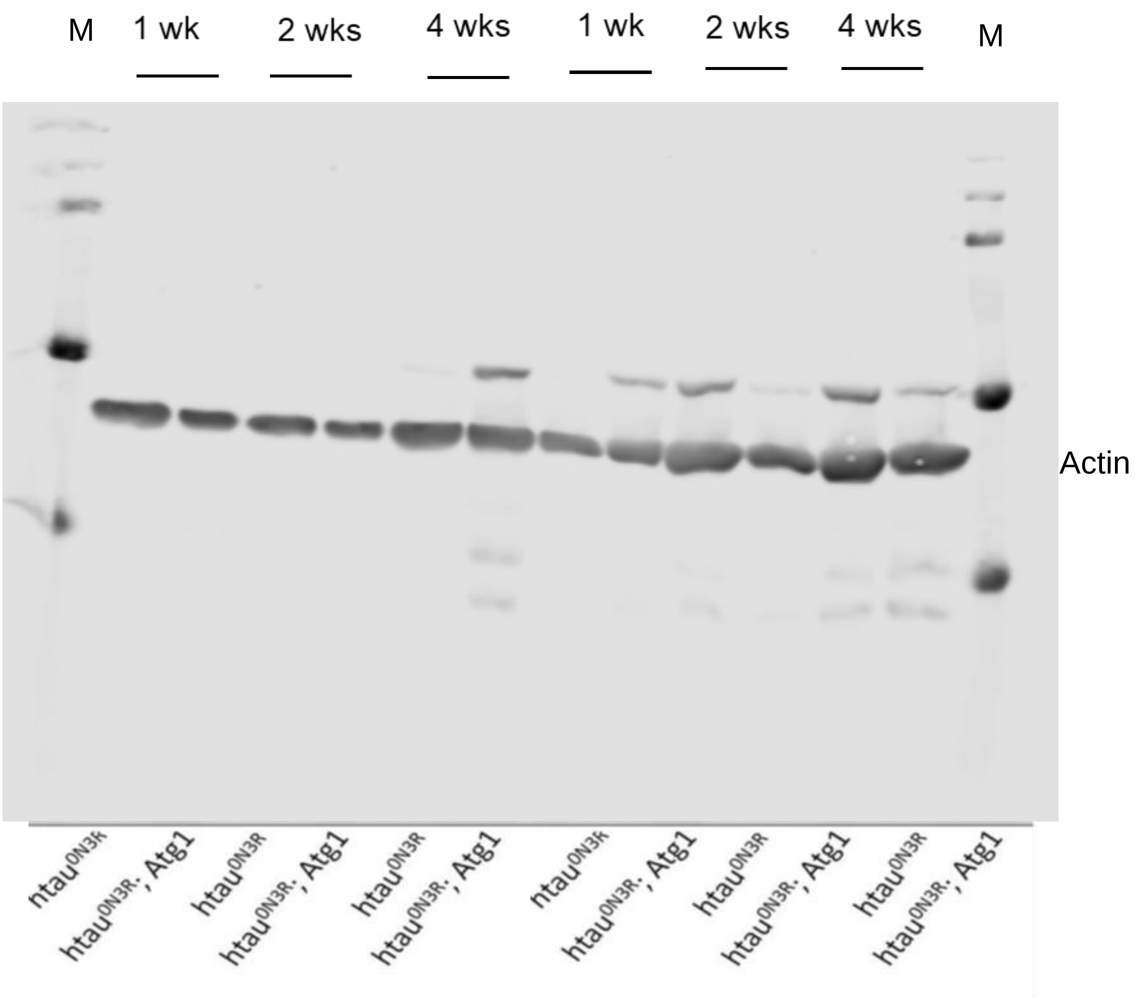

SUPPLEMENTARY FIGURES

Fig. S2 **Uncropped Blots for AT8 and p-TauS262 in Fig. S2**

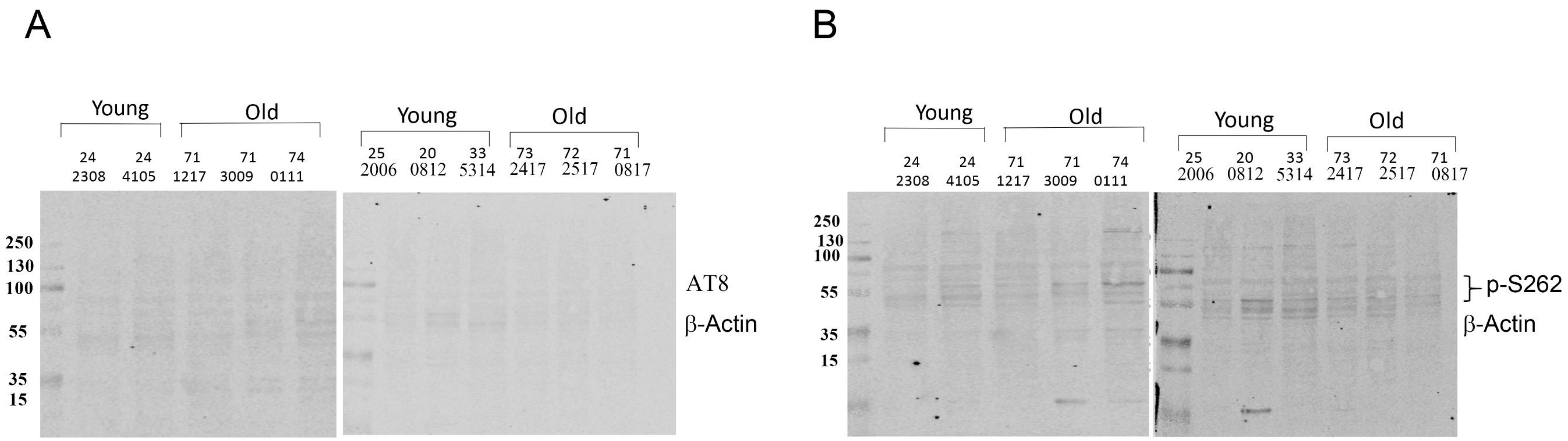

Supplementary Fig. S2:Phospho-tau immunoreactivity (AT8 and phospho-S262) in the Post-mortem brain samples was low. Representative Western Blot images for AT8, p-s262 and β-actin in post-mortem brains (A and B).There was no significant difference in the AT8 and p-s262 immunoreactivities between the younger vs older cohorts

Uncropped Blot for Tau isoforms Figure S3

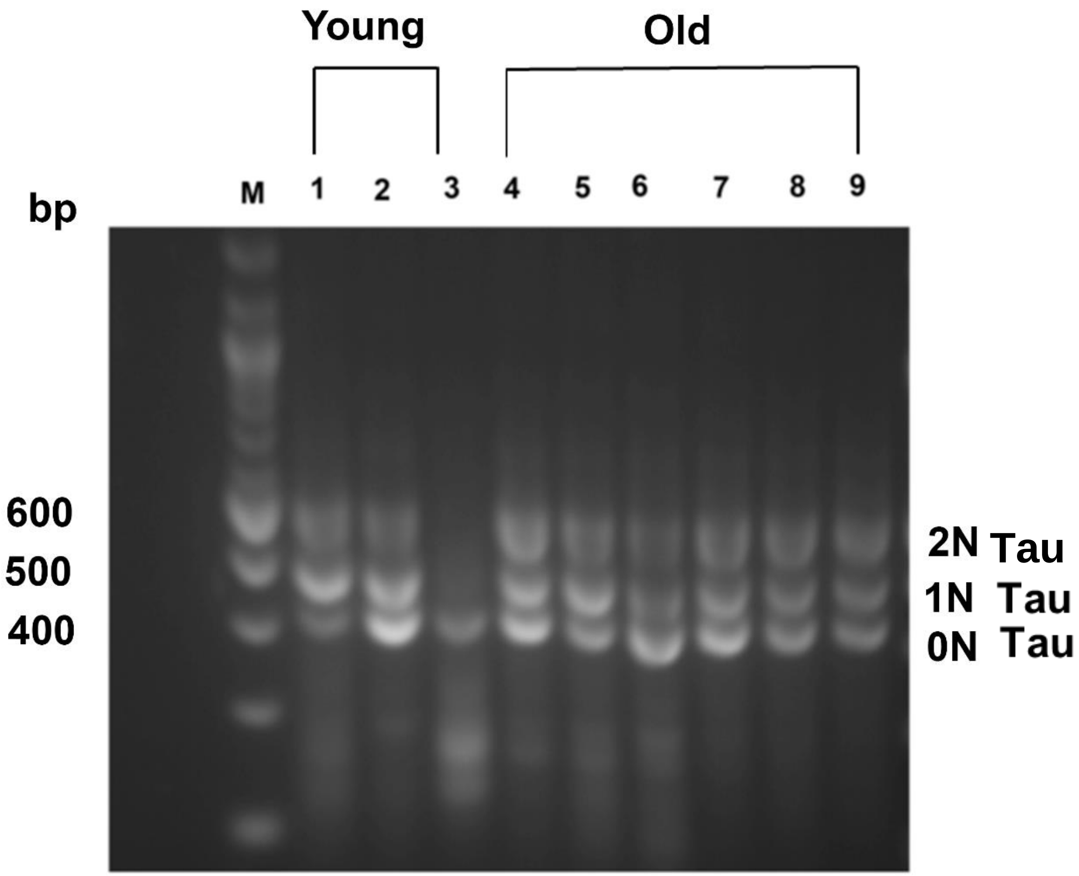

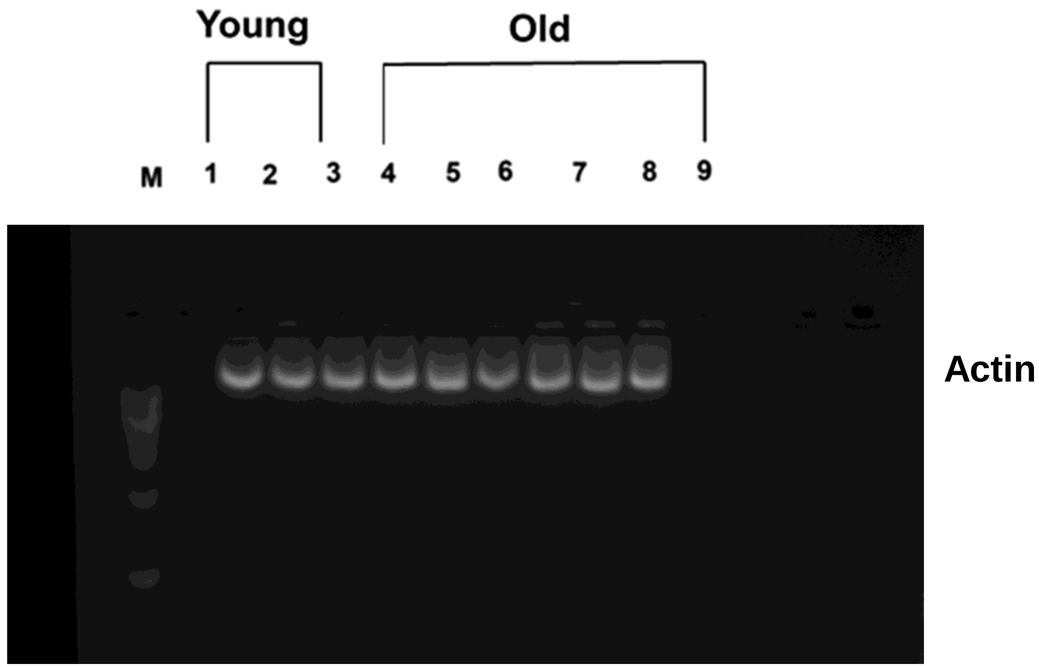

Uncropped blots for Cathepsin D and Actin in Fig. S4

Fig. S4

A

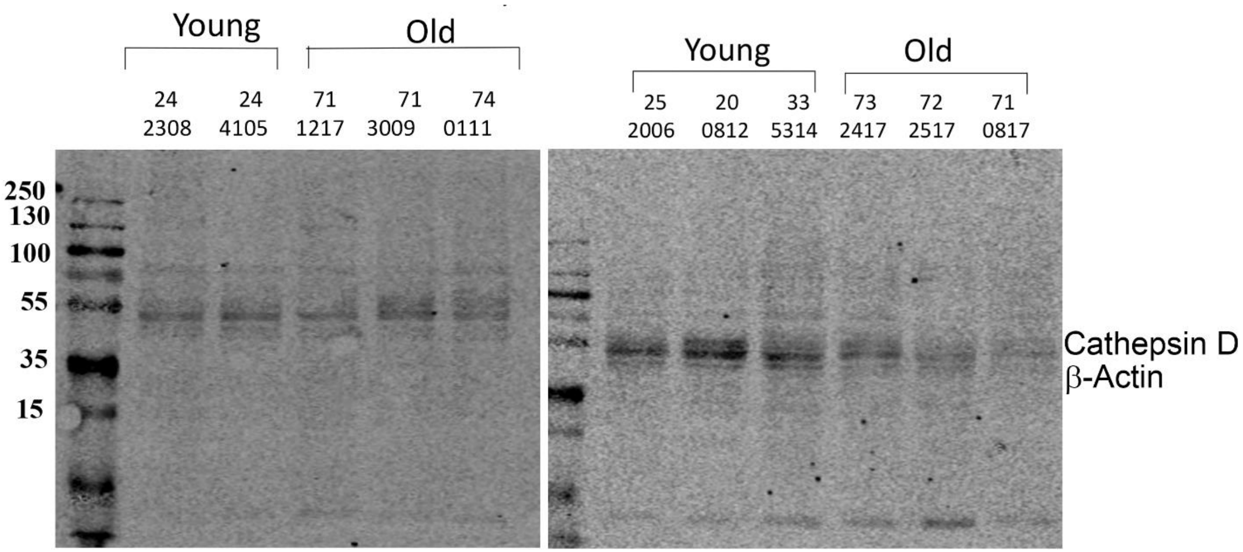

B

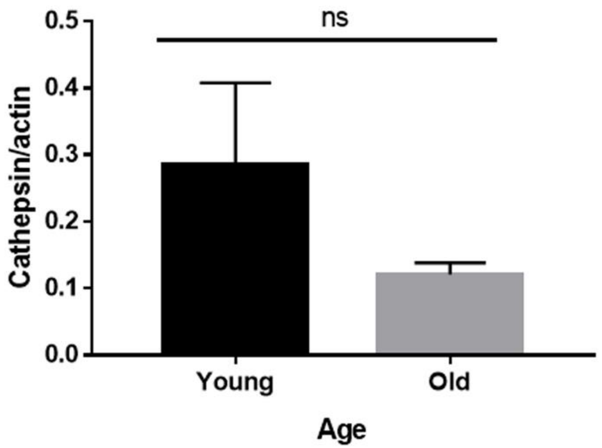

Supplementary figure 4 : Expression of the lysosomal protease Cathepsin D was not significantly different between the younger and older cohorts in the post-mortem brain tissues. (A) Representative Western Blot probed with Cathepsin D and β-actin loading control. (B) Quantitation of Cathepsin D normalised to β-Actin displays no significant difference.

Set 1, 2 and 3 – Uncropped Blot for Total hTau in Drosophila lines in Fig. S5A

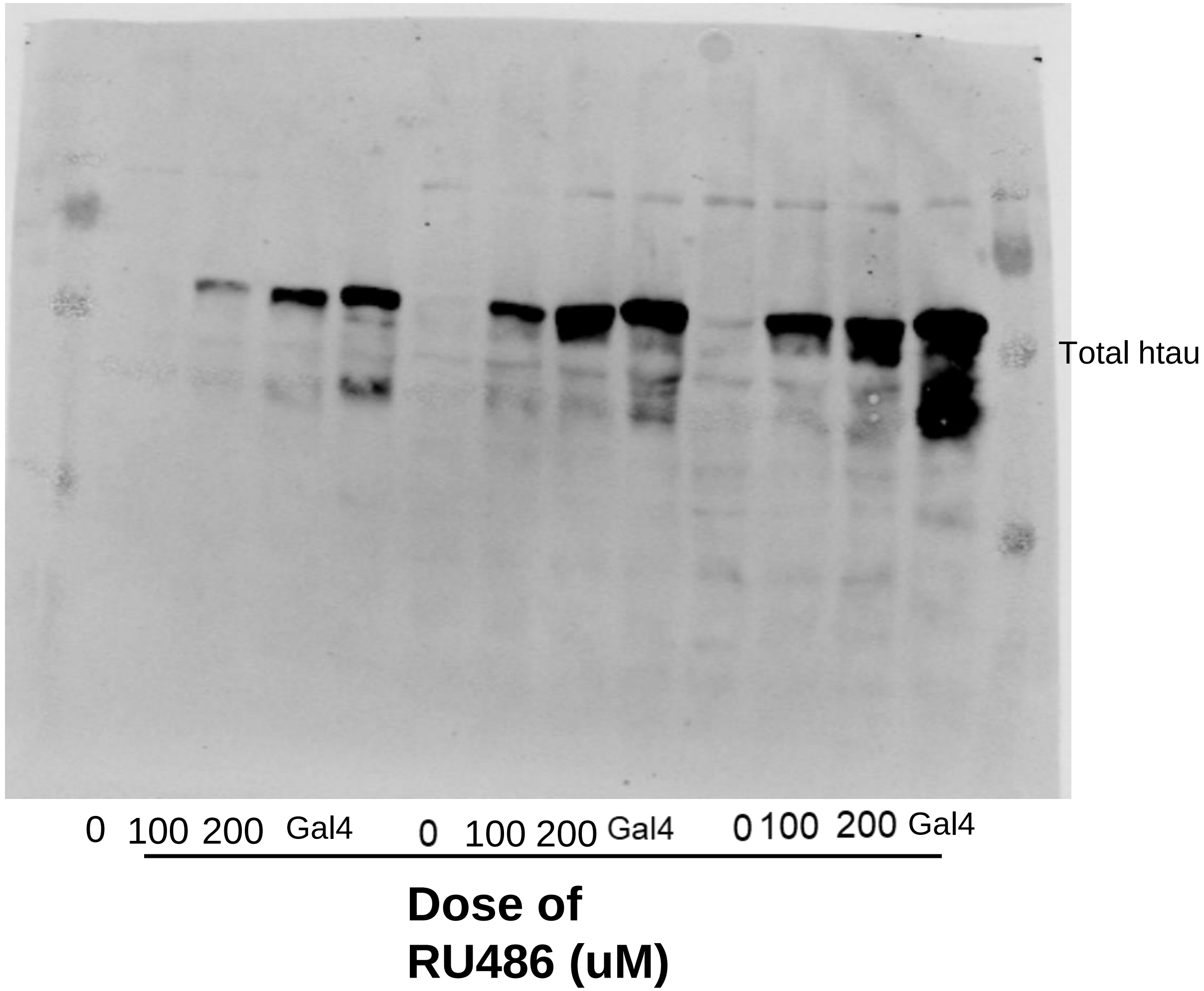

Uncropped blots for Atg8 and Actin for Fig. S5B

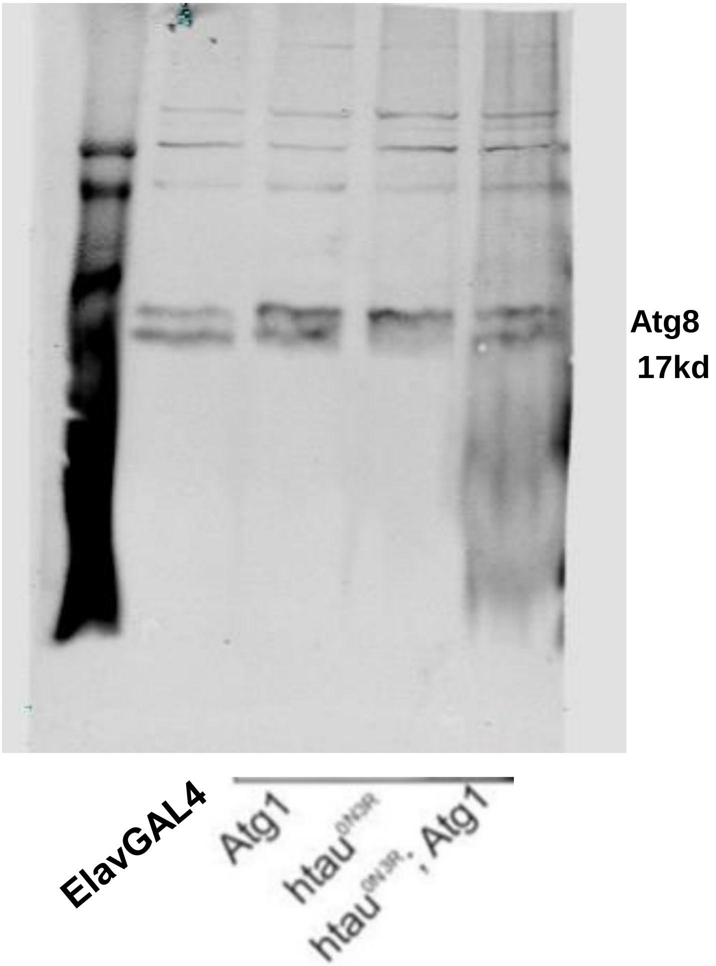

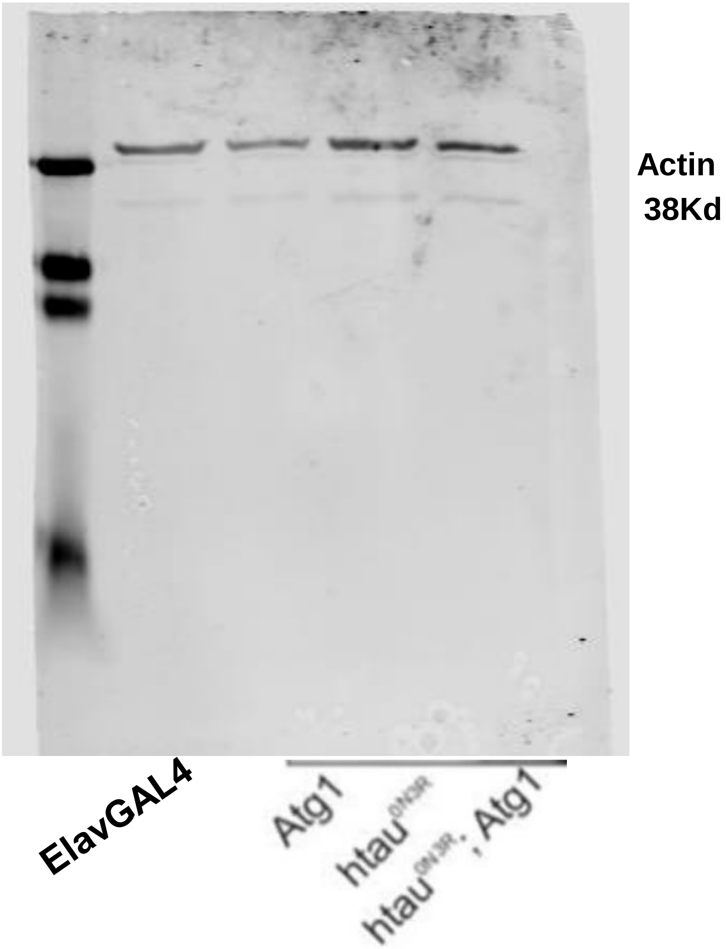

Uncropped blots for dTau and Kinesin in Fig.S6

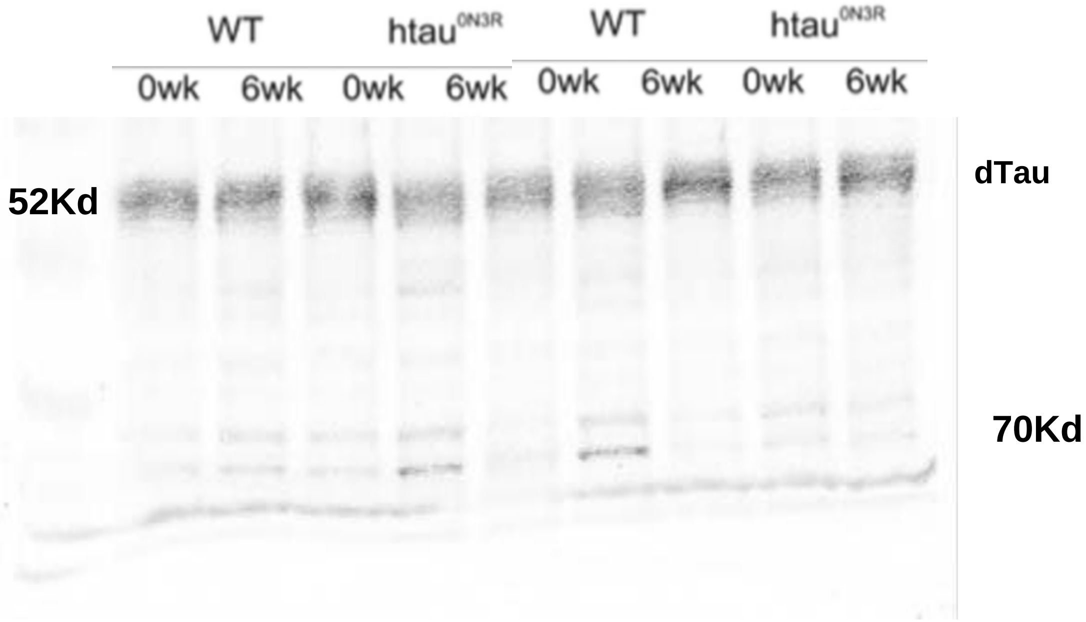

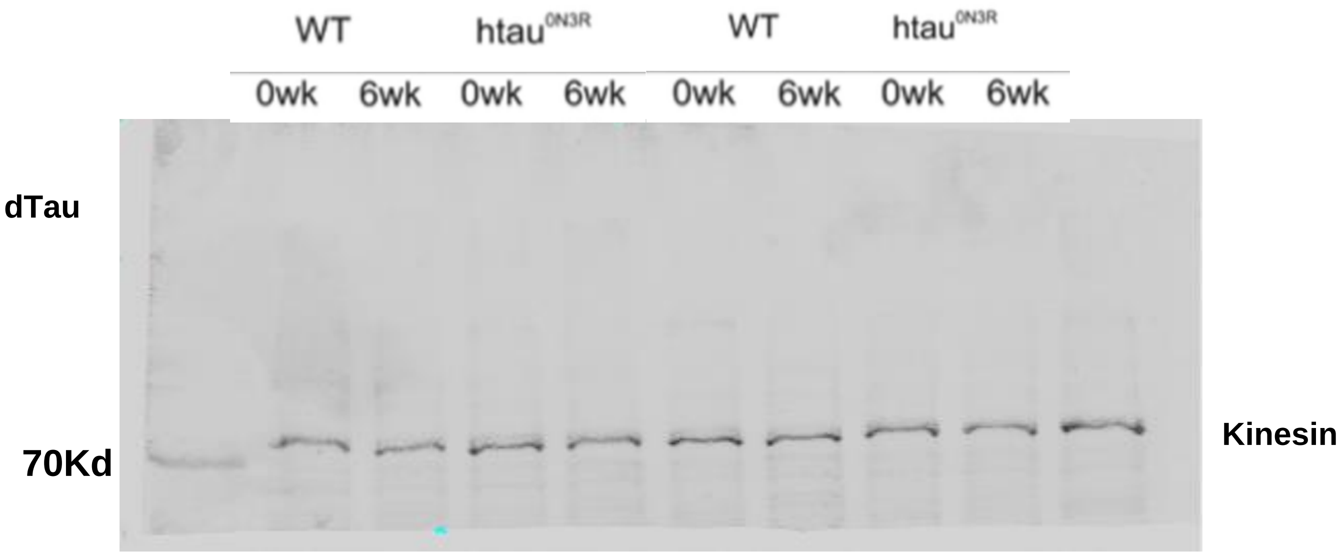

Supplement: S1 Raw images — (PDF) [file pone.0262792.s008.pdf]
