# Supplementary material for: Age-related changes in tau and autophagy in human brain in the absence of neurodegeneration
Source: PLoS One. 2023 Jan 26;18(1):e0262792. doi: 10.1371/journal.pone.0262792 (PMC9879510; doi:10.1371/journal.pone.0262792)
Supplement: Attachment — Submitted filename: Response to Reviewers.docx [file pone.0262792.s009.docx]

Reviewer #1: The authors address these questions by investigating physiological age-related changes in both tau and autophagy in human brain tissues taken from young and old subjects. The results seem to be very important in the research area of neurodegenerative diseases including Alzheimer’s disease. However, I have some points to clarify:

1. The authors showed that the levels of tau protein were reduced in old samples compared to young ones. In this study, they concluded that it was resulted from an increase in autophagy; however, it could be stemmed from the transcriptional repression of tau gene in the old samples. The authors should check mRNA levels of tau using qRT-PCR or RT-PCR.

We agree with the Reviewer. We have now done the RT-PCR experiments checking the tau mRNA levels and found no significant difference between the young and elderly cohorts (Suppl. Fig. 3). This has now been discussed in the results section (Page 12) and in the Discussion section (Page 20).

2. The authors insist that it is unclear whether autophagy contributes to the age-related reduction in soluble tau in the Discussion section (23 page). In autophagy-mediated degradation of tau, lysosomal activity is also critical to its degradation. So, authors did examine the levels of lysosomal enzymes such as ATG9b, cathepsin D, and CLCN7 and so on. To improve I recommend authors to examine those protein levels, at least cathepsin D.
We agree with the Reviewer. As recommended, by the Reviewer we have compared the levels of Cathepsin D between the young and old Post-Mortem brain samples and found no significant difference (Suppl. Fig. 4).

This has been included in the Results section (Page 16) discussed in Page 21.

3. Minorly, In the Discussion section, authors described that “With increasing age, a decrease in total tau levels and an increase in expression of two markers of autophagy were evident in the absence of overt neurodegeneration”. The expression seems to give readers a confusion. What are two autophagy markers increased in old samples? LC3II and p62; however, p62 was decreased.

We agree with the Reviewer. We have now changed this statement in the Discussion (Page 18).

Reviewer #2: This is an interesting and well-conducted study on the relation between total tau in old and young human cortex as well as a model system (Drosophila). However, there are a few issues that have to be addressed and that I list below.
1. On page 19 (of the PDF) the authors state that there is a consensus that there is no age-related decline in autophagy in brain : 1. the authors have to cite various appropriate references backing their statement and 2. I don't think that this consensus really exists as other studies/paper clearly demonstrate a decrease and I would rather recommend to state that this topic is rather controverse and surely this was one reason that the authors performed the current study.

The Reviewer is correct. This is indeed a controversial topic and this was one of the reasons to perform this study. We have highlighted this issue more strongly in our Introduction section on pages 4 and 5 as well as in the Discussion.

2. Please briefly describe what type of tau hTau0N3R is and what pathology it has in humans.

Human Tau in the brain has 6 isoforms with N-terminal and C-terminal repeats. hTau0N3R is the form of human Tau with no N-terminal but 3 C-terminal repeats. This 3-repeat Tau causes Pick’s Disease in humans. This has been included in the Results section on Page 16.

3. It seems that the authors only used one single phosphorylation site/antibody (PHF1) in their study. I am not sure that, considering the many different sites, analysing just 1 is really sufficient to confirm that tau in their brains is really hyperphosphorylated/pathological. The authors should make a strong case and discuss this issue, possibly as a limitation of their study.

We agree with the Reviewer. Due to the limited amount of the resected tissue only one antibody could be prioritized and we performed Western blot with PHF1 in this study. This limitation of our study has been discussed in the results and discussion. However, as we had more Post-Mortem tissues, based on the recommendation of the Reviewer we have now tested two other disease epitopes AT8 and phospho-serine 262 that are prominent in the brain of Alzheimer’s Disease patients. As with PHF1, we have not detected any immunoreactivity with the antibodies in the post-mortem brain tissues possibly due to the PM interval (Suppl. Fig. 2A and B). The results have now been updated on Page 12 and has been discussed on page 20 of the revised manuscript.

4. Importantly, the authors analyse only total and 1 phospho-tau site, but not aggregated tau which is the most likely cause for neurodegeneration compared to total tau. Autophagy is predominantly known to digest aggregated and misfolded proteins while soluble or oligomeric forms are rather degraded by other protein degradation mechanisms such as the proteasome. How do the authors exclude any influence of the latter on tau levels and why was aggregated tau not analysed? In the discussion decrease of soluble tau is briefly discussed as a speculation and aggregated mentioned, but the different forms of tau have to be explained in the introduction and reasons should be given why aggregated tau which can occur in non-demented older brains, has not been investigated.

We appreciate the Reviewer’s comments. The reason that aggregated tau was not studied is because the original paper on which our current study has been based showed that the reduction in soluble tau could not be explained by a correlative increase in insoluble tau in all the 5 regions of the brains that they examined. Our data supports the observations from Ladinska et al (Ref18). We have now bolstered the highlighted sections in the Results to justify why aggregated tau was not studied in the manuscript (Page 21).

5. Kang et al., 2019 (JAD) have demonstrated that autophagy activation participates in the secretion and transfer to neurons from both total and phospho-tau. Please include and discuss this novel observation into your manuscript.

We appreciate the Reviewer bringing the Kang et al. publication to our attention and we have now included the observations from this paper in our Discussion on Page 25 and added this publication to our reference list.

6. On page 1 of the introduction between references 7 and 8 suddenly high numbers (59, 65, 80 etc) appear. Similarly, on the next page suddenly reference 36 appears after 11 which is not correct. These reference numbers have to be integrated and give appropriate numbers as references should be numbered consecutively as they appear in the text.

We thank the Reviewer for bringing this to our notice. The Reference list has now been rectified and updated.

7. Additionally, "Pegasiou et al., 2020) in the results part needs to be transformed into a number and integrated into the consecutive numbering system.

Thank You. This has now been rectified.

8. Please explain "eclosion" as it is rather specific for Drosophila and not every reader is an expert in this area.

Eclosion in this case refers to the emergence of the adults from the pupal stage in the life-cycle of the *Drosophila melanogaster*. Based on the recommendation, we have now included “emerged” within brackets in our Results section on page 17 of our Results section.

9. What value does it have to analyse and show just a single case regarding electrophysiology?

We used electrophysiology in this case to justify that the neurons in the Resected tissue samples were physiologically active at the time of collection. This has been clarified in the Results section on Page 10.

10. There is no colour in suppl. fig. 4, please add since otherwise everything looks just grey. If no colour should be used then discriminate the lines/curves differently (using dashed lines, for example.

The suppl. Fig 4 has now been changed to suppl. Fig.7 and modified based on Reviewer’s comments.
